# Laundering and evasion in response to antislavery policies in Brazil’s cattle sector

**DOI:** 10.1073/pnas.2532121123

**Published:** 2026-10-12

**Authors:** Tara Mittelberg, Marin E. Skidmore, Juliana L. S. C. Brandão, Lisa L. Rausch, Holly K. Gibbs

**Affiliations:** ^a^ Nelson Institute for Environmental Studies, Center for Sustainability and the Global Environment, University of Wisconsin–Madison, Madison, WI 53726; ^b^ Department of Agricultural and Applied Economics, University of Wisconsin–Madison, Madison, WI 53706; ^c^ Department of Agricultural and Consumer Economics, University of Illinois at Urbana-Champaign, Urbana, IL 61801

**Keywords:** modern slavery, supply chains, agriculture, public policy

## Abstract

Modern slavery remains pervasive across many economic sectors. Progress toward eradicating it has been partially hindered by a lack of tested, comprehensive policy responses. Using data on millions of cattle transactions in Brazil, we evaluated how ranchers changed their selling behavior after being added to the “Dirty List,” a public registry that names and shames employers found to use modern slavery. Dirty Listed ranchers significantly reduced direct sales to slaughterhouses that signed sustainable supply chain commitments but evaded sanctions by laundering cattle through compliant intermediaries or selling to nonsignatory slaughterhouses. These responses weakened the financial cost of listing to ranchers, and likely allowed beef and leather linked to modern slave labor to enter international supply chains. Our findings highlight the limits of downstream enforcement and underscore the need to monitor upstream suppliers to ensure slavery-free supply chains.

## Significance

Governments and companies have worked to rid supply chains of modern slavery, but little is known about policy outcomes, including how perpetrators change their behavior in response to sanctions. With data on millions of cattle transactions in Brazil, we present rigorous empirical evidence that employers implicated in modern slavery systematically evaded supply chain policies. Ranchers on the government’s “Dirty List” for forced labor ceased sales to slaughterhouses that signed supply chain commitments, but simultaneously increased sales to other ranchers who then sold cattle to the same slaughterhouses. As a result of this cattle laundering, supposedly clean domestic and international supply chains were contaminated with modern slave labor. Comprehensive monitoring and enforcement are necessary to ensure clean supply chains.

Nearly 30 million people worldwide are currently subject to forced labor, an illicit and pernicious form of modern slavery that persists in supply chains ranging from mining to seafood to readymade garments (1). Supply chain experts agree that a combination of policies—including strong public enforcement, transparency measures, and comprehensive private-sector supply chain sanctions—are necessary to ensure slavery-free supply chains, especially in industries with many dispersed suppliers (2, 3). However, data constraints stemming from the hidden nature of modern slave labor (MSL) have prevented rigorous evaluation of anti-MSL policies and whether they achieve truly clean supply chains.

The cattle sector is a major perpetrator of labor exploitation in Brazil, accounting for more than a quarter of the 65,000 workers ever rescued from MSL (4). Brazil’s penal code defines “conditions analogous to slavery” as those that subject a worker to arduous working days, degrading working or living conditions, restricted movement, or debt bondage (5). The Ministry of Labor maintains the “Dirty List,” a registry of employers that have been found to have used MSL. The list is available online and is updated every six months. Dirty Listed employers are removed from the list after two years as long as they have paid relevant fines and passed a labor inspection. Brazilian courts have emphasized that the Dirty List is a transparency mechanism and not a sanction (6). Listed employers cannot access credit from public banks, but no law explicitly prohibits private companies from conducting business with them (see *SI Appendix*, section S1 for more background information).

The business consequences of appearing on the Dirty List therefore largely depend on private-sector-led sanctions (7). Hundreds of companies across diverse sectors have pledged to boycott employers on the Dirty List (8). In the cattle sector, these include export-oriented meatpacking companies that signed one or both zero-deforestation Cattle Agreements, known as the “Termos de Ajustamento de Conduta da Carne” (Beef-TAC signed with Public Federal Prosecutors; here, just TAC) and the Public Livestock Commitment (PLC; formerly known as the G4 and signed with Greenpeace). While participation in the PLC is fully voluntary, companies that sign the TAC sometimes do so based on the threat of a lawsuit by prosecutors, and once the agreement is signed, it is legally binding. Together the agreements cover 75% of all export-oriented slaughterhouses in the Amazon. They both include provisions that prohibit purchasing animals from Dirty Listed ranchers or from those with deforestation or other environmental infractions (9, 10). The PLC companies—which include the three largest meatpackers that collectively slaughter more than half of the cattle in the Amazon—have been the most active in implementing the Cattle Agreements and are under the most scrutiny due to their prominent role in international supply chains.

**Fig. 1.**
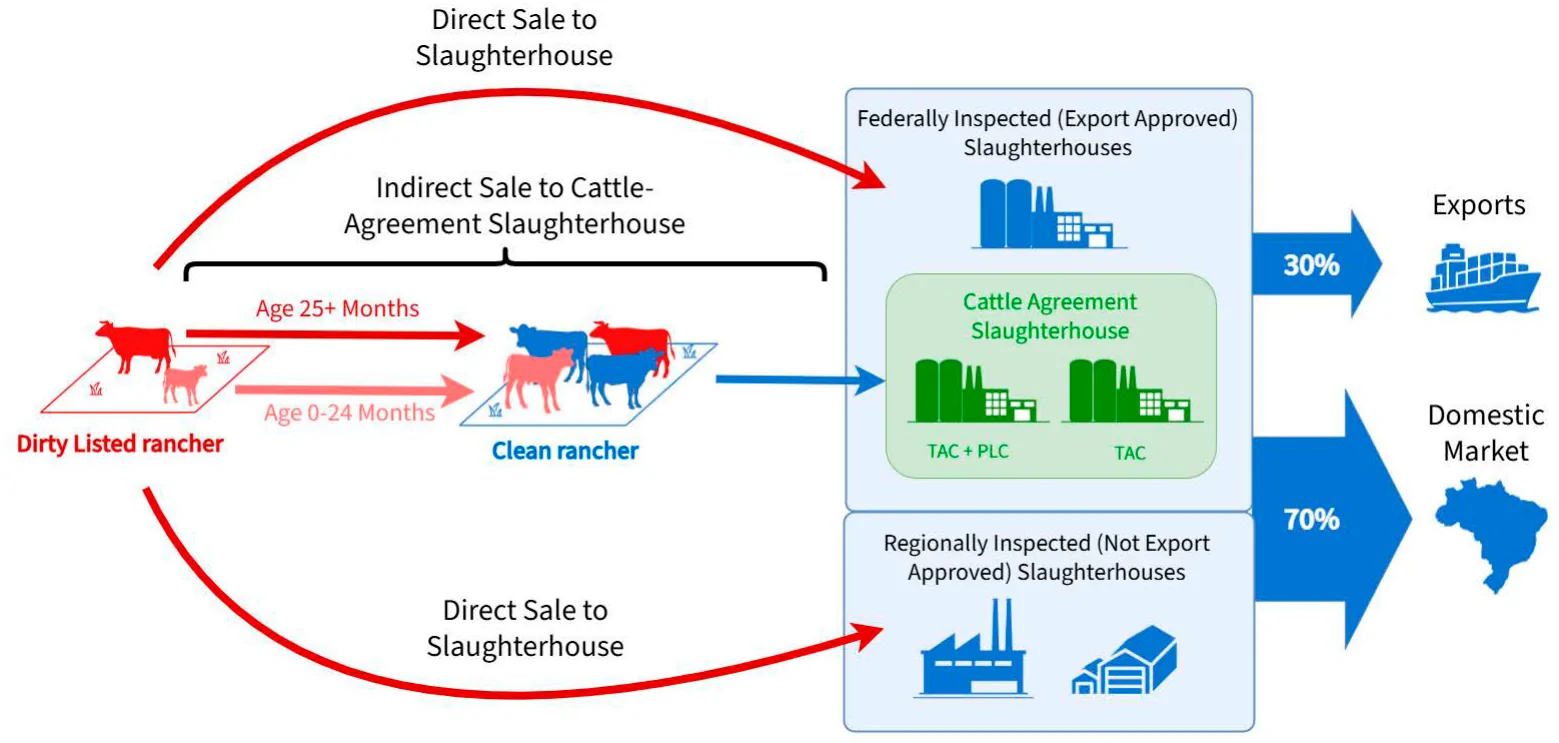
The cattle supply chain. We tested how ranchers changed sales to various destinations while on the Dirty List. “Direct Sales” are those to slaughterhouses for the purpose of slaughter. We disaggregated direct sales by slaughterhouse type (level of inspection and whether it signed the PLC or TAC Cattle Agreements). “Indirect Sales” are those to other ranchers. We disaggregated indirect sales by the age of the animals involved in the transaction to assess whether ranchers switched to earlier stages of the production process while listed, or continued to sell mature, slaughter-age animals into supply chains. To assess whether cattle raised by Dirty Listed ranchers likely reached Cattle-Agreement supply chains, we also specifically tested for changes in indirect-PLC and indirect-TAC sales, which are sales of slaughter-age animals to another rancher who subsequently sold to a PLC or TAC slaughterhouse. We then assessed whether cattle raised by Dirty Listed ranchers likely ended up in international supply chains. On average, 30% of cattle products produced in Brazil are exported (11).

We evaluated whether the combination of Brazil’s public Dirty List and private sector sanctions allowed companies to rid supply chains of goods produced by labor-exploiting employers, and assessed the economic consequences for listed ranchers. To do so, we used a dataset of millions of cattle transactions in the Brazilian Amazon and a staggered difference-in-differences and event study design to estimate changes in ranchers’ sales behavior before, during, and after they appeared on the Dirty List. Our detailed transaction-level data allowed us to trace not only changes in total cattle sales when a rancher was on the Dirty List, but also shifts across destination types, revealing how ranchers strategically adjusted their roles in the supply chain in response to sanctions (Fig. 1).

First, we evaluated whether listed ranchers reduced their direct sales to Cattle Agreement slaughterhouses during and after listing, an indication of whether these buyers successfully monitored and blocked noncompliant suppliers in fulfillment of their sustainable supply chain commitments. For comparison, we also estimated changes in sales to slaughterhouses that signed neither agreement but are export-oriented and inspected by federal authorities,^*^ and are therefore comparable to Cattle Agreement signatories; we also estimated changes in sales to smaller, local-market slaughterhouses that signed neither agreement. To assess whether listed ranchers continued to sell cattle despite being blocked by slaughterhouses, we then estimated changes in sales to other ranchers, which we call indirect sales. We separately estimated changes in indirect sales of younger animals (<24 mo) and slaughter-age animals (≥24 mo) to evaluate whether blocked ranchers shifted their supply chain position to earlier stages (rearing and breeding), or continued to sell fattened, slaughter-age animals into supply chains via clean ranchers. To assess risk of contamination of supposedly clean supply chains, we estimated changes in indirect-PLC and indirect-TAC sales, defined as sales of slaughter-age animals to other ranchers who then sold to Cattle Agreement (PLC or TAC) slaughterhouses within one year. In absence of animal-level traceability data, this measure provides the closest proxy of whether Dirty Listed ranchers indirectly sold cattle to Cattle Agreement slaughterhouses, capturing sales of slaughter-age animals into herds that subsequently supplied these buyers. Finally, we estimated changes in total head sold, an indicator of how the Dirty List impacted the overall output of ranchers during listing and after being removed.

Our results show that despite being blocked by Cattle-Agreement slaughterhouses, Dirty Listed ranchers continued to access supply chains by selling to nonsignatory slaughterhouses or rerouting cattle through compliant intermediaries to reach Cattle-Agreement slaughterhouses. These tactics led to contamination of supposedly clean supply chains and reduced the economic impacts of sanctions for listed ranchers. To better understand the consequences of these behavioral changes, we conducted two complementary analyses. First, we analyzed shipment-level export data from a subset of our study years to determine how slaughterhouses that purchased cattle from Dirty Listed ranchers were connected to international supply chains. We also estimated the financial costs of shifting from direct to indirect access to Cattle Agreement slaughterhouses for blocked ranchers. Our study covers 2009–2023 and focuses on the Brazilian Legal Amazon states of Pará, Mato Grosso, Rondônia, and Tocantins, which together account for 36% of individuals ever rescued from modern slavery in Brazil (4).

We present causal tests that track how employers sanctioned for using MSL respond to and circumvent antislavery policies. We link modern slavery in upstream suppliers in Brazil’s cattle sector to domestic and international supply chains. Ridding supply chains of MSL will require sector-wide monitoring and stringent exclusion mechanisms that are difficult to evade.

## Results

### Dirty Listed Ranchers Reduced Sales to Cattle-Agreement Slaughterhouses.

**Fig. 2.**
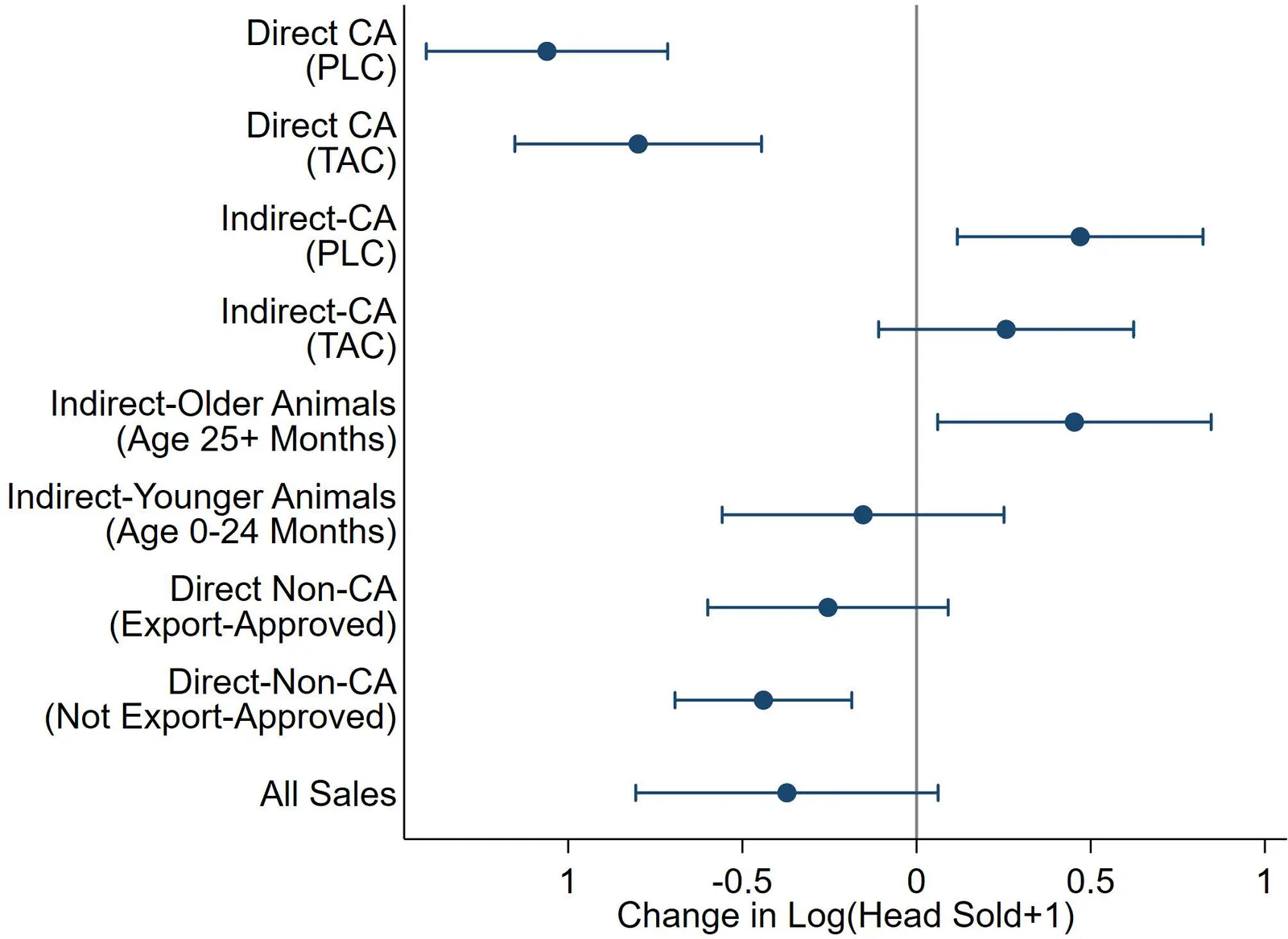
Change in head sold when a rancher is on the Dirty List, by sales destination, compared to prelisting levels. While on the Dirty List, ranchers cut nearly all direct sales to slaughterhouses that signed the TAC and PLC cattle agreements (CAs). However, they significantly increased sales of slaughter-age animals to other ranchers who subsequently went on to sell animals to TAC and PLC slaughterhouses, suggesting that they laundered cattle through clean ranches to indirectly access Cattle-Agreement slaughterhouses. Dirty Listed ranchers did not significantly change sales behavior toward export-approved non-Cattle Agreement slaughterhouses. Bars represent 95% CIs. Results are from the primary specification (OLS with rancher and period fixed effects) and correspond with results from Column 1 of Table 1. Estimation sample is 6,502 rancher-semesters and includes only ranchers who were ever on the Dirty List.

**Fig. 3.**
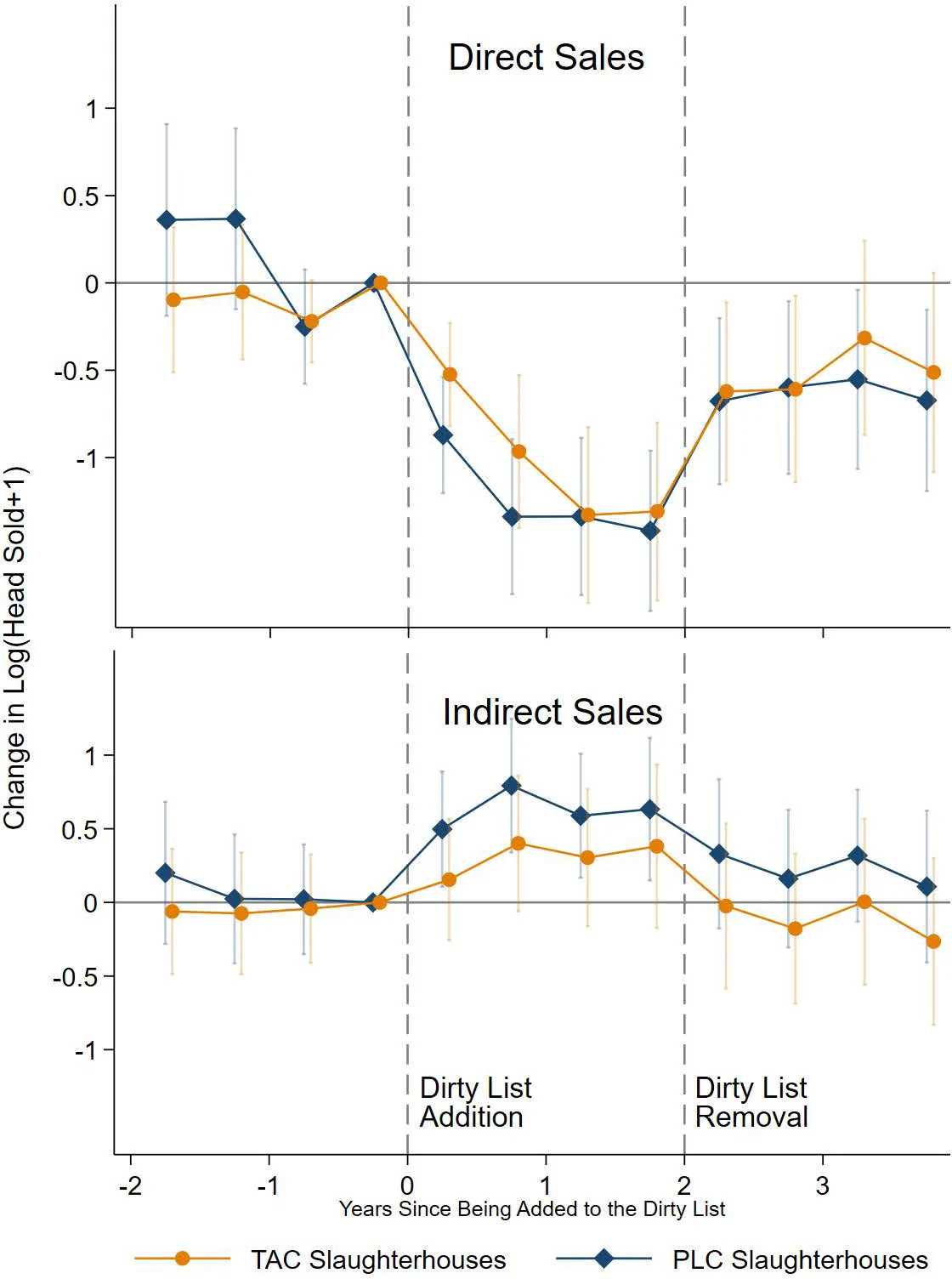
Semesterly changes in head sold to cattle-agreement (PLC and TAC) slaughterhouses before, during, and after appearing on the Dirty List. Ranchers reverted toward business as usual with slaughterhouses that signed the PLC and/or TAC cattle agreements within six months of being removed from the Dirty List, though did not return to prelisting levels. Indirect sales behavior reverted to baseline levels after being removed from the list, suggesting that ranchers strategically altered sales behavior while sanctioned. Bars represent 95% CIs. Event study model estimated with the Chaisemartin-D’Haultfæuille estimator with rancher and semester effects (29). Reference period is one semester prior to listing.

Compared to prelisting levels, ranchers on the Dirty List reduced direct sales to Cattle-Agreement slaughterhouses by more than half (PLC sales: −65.4%, 95% CI = [−75.7%, −50.7%]; TAC sales: −55.1% [−68.4%, −36.1%]) (Fig. 2). At the mean level of sales, this amounted to an average per-ranch reduction of 176 head per year to PLC slaughterhouses and 191 head per year to TAC slaughterhouses. These effects persisted throughout the two years a rancher was on the Dirty List (Fig. 3). After being removed, sales to TAC slaughterhouses returned to business-as-usual (−28.1% [−53.3%, 10.6%]), while sales to PLC slaughterhouses increased compared to the period a rancher was on the Dirty List, but did not fully recover to prelisting levels (−44.0% [−60.7%, −20.3%]) (Table 1).

**Table 1.** Changes in cattle sales to different destinations during and after dirty listing

Outcome (Log(Head Sold+1))	Rancher on DL	Rancher formerly on DL	*P*-val: On DL = Formerly on DL	Obs	$R^{2}$	Mean sales (head per semester)
All sales	−0.37∗ (0.22)	−0.64∗∗ (0.28)	0.17	6,502	0.608	943
Direct sales only						
All direct sales	−1.26∗∗∗ (0.21)	−1.17∗∗∗ (0.23)	0.64	6,502	0.617	323
Direct CA - PLC	−1.06∗∗∗ (0.18)	−0.58∗∗∗ (0.18)	0.00∗∗∗	6,502	0.550	135
Direct CA - TAC	−0.80∗∗∗ (0.18)	−0.33 (0.22)	0.00∗∗∗	6,502	0.553	173
Direct non-CA - SIF	−0.25 (0.18)	−0.46∗∗ (0.21)	0.13	6,502	0.414	22
Direct non-CA - Non-SIF	−0.44∗∗∗ (0.13)	−0.54∗∗∗ (0.14)	0.33	6,502	0.409	66
Indirect sales only						
All indirect sales	0.21 (0.21)	−0.25 (0.26)	0.02∗∗	6,502	0.521	620
Indirect - Younger (0 to 24 mo)	−0.15 (0.21)	−0.10 (0.27)	0.77	6,502	0.482	302
Indirect - Older (24+ mo)	0.45∗∗ (0.20)	−0.05 (0.24)	0.00∗∗∗	6,502	0.430	275
Indirect CA - PLC	0.47∗∗∗ (0.18)	0.14 (0.17)	0.02∗∗	6,502	0.382	113
Indirect CA - TAC	0.26 (0.19)	−0.09 (0.21)	0.02∗∗	6,502	0.418	176

SEs in parentheses. Estimated coefficients represent average log changes in head sold to a given destination while a rancher was on the Dirty List or formerly listed, compared to the period when they were not-yet-listed. Every row represents the coefficient of interest from a separately estimated model. “Rancher on DL” is a dummy equal to one if the rancher was on the Dirty List during period *t*. “Rancher Formerly on DL” equals one if the rancher in period *t* was previously on the Dirty List but had been removed. *P*-values are from an F-test of equivalence between coefficients presented in that row (i.e. whether the average rancher’s sales to that destination were the same in the periods when they were on the Dirty List compared to the periods after they were removed). Model estimated with OLS with rancher and period fixed effects on the sample of ever-listed ranchers. ∗ P<0.10, ∗∗ P<0.05, and ∗∗∗ P<0.01.

We found a smaller decrease in direct sales to slaughterhouses that did not sign the Cattle Agreements. Relative to prelisting levels, ranchers did not significantly change sales to non-Cattle-Agreement slaughterhouses that were large, federally inspected, and certified for export (−22.1% [−45.3%, 10.8%]). Ranchers sold 35.6% fewer cattle to smaller, locally inspected non-Cattle-Agreement slaughterhouses while on the Dirty List [−50.1%, −16.9%]. Some ranchers may have shifted their strategy to focus on indirect sales while listed, thereby also decreasing direct sales to non-Cattle-Agreement slaughterhouses.

Notably, however, sales to non-Cattle-Agreement slaughterhouses did not stop altogether. Sales to non-Cattle-Agreement slaughterhouses represented approximately 27% of all direct head sold (176 head per rancher per year) in our study sample, providing an important alternative market and sizable buffer for sanctioned ranchers. Persistent opportunities to sell to non-Cattle-Agreement slaughterhouses have limited the effectiveness of the Cattle Agreements in curbing deforestation (12, 13), and may similarly hinder efforts to penalize MSL.

After being removed from the Dirty List, sales to non-Cattle-Agreement slaughterhouses remained lower than before listing (non-CA export-certified: −36.9% [−58.2%, −4.7%]; non-CA not-export-certified: −41.7% [−55.7%, −23.3%]). This result parallels relative changes in total sales, discussed below.

### Cattle-Agreement Supply Chains Still Contaminated by Modern Slave Labor.

Despite reductions in direct sales, Dirty Listed ranchers continued to sell indirectly to Cattle-Agreement slaughterhouses. Although Cattle-Agreement slaughterhouses assessed whether their direct cattle suppliers were on the Dirty List, they did not monitor their indirect suppliers, which include those ranchers involved in earlier stages of cattle production such as breeding and fattening, or those selling through intermediary ranches rather than directly to slaughterhouses (9).

While on the Dirty List, ranchers significantly increased sales of slaughter-age (>24 mo) cattle to other ranchers, many of whom subsequently sold to Cattle-Agreement slaughterhouses. On average, Dirty Listed ranchers increased sales of slaughter-age animals to other ranchers by 56.8% [6.0%, 132.1%], or 313 head per year. We further refine these sales by whether the buying rancher went on to sell to a cattle agreement (PLC or TAC) slaughterhouse within one year. Compared to prelisting levels, indirect-PLC sales increased by 60.0% [12.4%, 127.7%], or 136 head per year. Indirect sales linked to TAC slaughterhouses followed a similar pattern, although increases in indirect-TAC sales by Dirty Listed ranchers were only significant compared to the post-listing period, possibly reflecting the later roll-out of and greater heterogeneity in TAC implementation during the study period.^†^ Many of the cattle produced by Dirty Listed ranchers ended up in the same Cattle-Agreement slaughterhouses as they would have had the rancher been able to sell directly.^‡^ In contrast, Dirty Listed ranchers did not change the number of younger animals they sold to other ranchers (−13.9% [−43.0%, 29.9%]). This suggests that Dirty Listed ranchers strategically shifted their sales behavior toward indirect sales of market-ready cattle, effectively laundering animals through compliant intermediaries to maintain access to Cattle-Agreement supply chains.

Within six months of being removed from the Dirty List, ranchers increased direct sales to PLC slaughterhouses (though not to prelisting levels) and decreased indirect sales to PLC slaughterhouses to prelisting levels (Fig. 3). This return to business as usual suggests that ranchers and slaughterhouses adjusted their market relationships temporarily in response to policy constraints. It also suggests that ranchers value being able to sell directly to slaughterhouses due to a financial benefit or other business reasons.

### Total Sales Did Not Fully Recover After Dirty List Removal.

Though ranchers maintained access to the supply chain through indirect sales, they decreased total sales by 30.9% [−55.1%, 6.3%] during listing and by 47.3% [−69.5%, −8.7%] after listing relative to the levels prior to listing. These results indicate that appearing on the Dirty List potentially caused persistent shifts in ranchers’ production, likely driven by costs related to the administrative process of appearing on the Dirty List (such as transitioning to a compliant production system and paying legal fees and fines), and being excluded from the preferred supply chain. The implications of these production shifts are ambiguous and should be explored in future empirical or theoretical work. On one hand, the larger the short- and long-term effect of the Dirty List on sales, the greater its potential to deter MSL (see theoretical model in *SI Appendix*, section S4). On the other hand, the potential to resume normal activities may serve as an incentive for Dirty Listed ranchers to pursue and maintain compliant production practices during and after listing.

### Laundering Cattle Is Inexpensive for Sanctioned Ranchers.

Despite reductions in total sales, some ranchers laundered cattle while on the Dirty List, offsetting the costs of Cattle-Agreement exclusion. To provide a rough estimate of the cost of Cattle-Agreement exclusion for a rancher able to launder cattle, we estimated three main price penalties faced by a Dirty Listed rancher selling indirectly to Cattle-Agreement slaughterhouses compared to a rancher able to access Cattle-Agreement slaughterhouses directly (see *SI Appendix*, section S3.2 for details). First, transporting animals to a clean ranch costs approximately 0.8% (R$29 per animal) of the expected revenue compared to selling directly to a Cattle-Agreement slaughterhouse. Another potential cost is a fee taken by the clean rancher to launder cattle. Buyers who serve as intermediaries for different types of cattle sales in our study area typically collect commissions ranging from 1 to 14% of the value of the cattle sold. However, Dirty Listed ranchers can avoid transportation costs and intermediary commissions by simply filing an additional cattle movement (GTA) form in the name of a willing clean rancher, meaning the only guaranteed cost of laundering is the paperwork filing fee, a maximum of R$4.72 per animal, or 0.1% of the expected revenue of selling cattle directly to a Cattle-Agreement slaughterhouse.

### Contaminated Cattle Likely Reached International Destinations.

Because Dirty Listed ranchers remained in the supply chains of major slaughterhouses, cattle linked to MSL likely ended up in both international and domestic markets. Of the slaughter-age animals that left Dirty Listed ranches, 75% were sold either directly to export-oriented slaughterhouses or to other ranchers who sold to those slaughterhouses within one year. In total, 241 slaughterhouses were connected to Dirty Listed ranchers through direct and indirect sales (Table 2). These transactions represented approximately 200,000 head slaughtered, 40% of which were purchased directly from Dirty Listed ranchers and the rest of which passed through another ranch, at least on paper, before being purchased by the slaughterhouse. Using export data from 2014, 2018, and 2019, we identified at least 51 slaughterhouses that exported beef or leather products in the same year they bought from Dirty Listed ranchers. These slaughterhouses made up a sizable share of all beef and leather exporters in our dataset (21% of those in our study states). Export destinations for these sales included the European Union, United Kingdom, and China (*SI Appendix*, Table S5). Although data limitations prevented us from tracing export volumes or sales destinations for the entire study period, our results show that gaps in supplier monitoring allow products linked to MSL to enter both domestic and international supply chains.

**Table 2.** Number of slaughterhouses connected to dirty listed ranchers

	CA- PLC	CA- TAC	Non-CA EC	Non-EC Non-CA	Total
Bought from Dirty Listed rancher directly or indirectly					
Total	35	90	15	154	241
Exported at any point in study period	32	51	1	1	57
Exported in same year as dirty purchase	28	47	1	1	51
					
Bought from Dirty Listed rancher directly					
Total	15	54	9	74	121
Exported at any point in study period	15	29	0	0	30
Exported in same year as dirty purchase	6	15	0	0	15
Bought from Dirty Listed rancher indirectly					
Total	35	87	12	119	201
Exported at any point in study period	32	51	1	1	57
Exported in same year as dirty purchase	28	47	1	1	51

Note: “EC” = Export Certified (federally inspected) slaughterhouses. “CA” = Cattle Agreement. To avoid double-counting slaughterhouses, for the export analysis we did not account for the fact that many export-certified slaughterhouses switched from being non-CA to being TAC signatories during our study period. Thus, here, “TAC” refers to slaughterhouses that ever signed the TAC, while “Non-CA” refers to slaughterhouses that never signed either CA. This differs from our approach in the main causal analysis, where we are able to code each transaction as non-CA or TAC based on whether the slaughterhouses had signed the TAC at the transaction time. This simplification in the export analysis means that actual TAC slaughterhouse numbers are potentially lower than those presented in this table, and actual non-CA EC numbers are potentially higher. All export-related counts are almost certainly higher, since export data only cover 2014, 2018, and 2019, while the study period spanned 2009–2023.

## Discussion

### Study Limitations.

One limitation of our study is reliance on the public Dirty List to determine which ranchers perpetrated MSL. As a result, our sample of ranchers only includes those who were caught and appeared on the Dirty List, which is known to greatly underestimate the extent of labor exploitation (14); similarly, we could not determine whether the combination of the Dirty List and Cattle Agreements deterred MSL.

Another limitation is that our supply chain data do not allow us to track the movements of individual animals. This means that we were unable to determine the ultimate destination of the cattle that were raised by Dirty Listed ranchers, and instead approximated indirect sales to Cattle-Agreement slaughterhouses as those in which a rancher sold slaughter-age animals to another rancher who then sold to a Cattle-Agreement slaughterhouse within one year. It is unclear whether this approximation resulted in over- or underestimation of supply chain contamination. On one hand, we cannot guarantee that all of the cattle involved in these indirect sales indeed went to Cattle-Agreement slaughterhouses. On the other hand, this definition is limited in that it only includes first-tier indirect suppliers, meaning that we miss cases in which Dirty Listed ranchers were one or more supply chain links from clean ranchers who sold directly to slaughterhouses. Relatedly, we were unable to link records of live animals sold for slaughter to the beef and leather products that meatpackers sold into domestic or international consumer supply chains, and so cannot precisely quantify the level of contamination of different consumer markets.

Finally, we used a staggered difference-in-differences approach to estimate average changes in sales when a rancher was on the Dirty List or previously listed, compared to prelisting periods. Because our average effects model has two treatment periods (during- and post-listing), we estimated it with OLS and two-way fixed effects, which may be vulnerable to bias in the presence of heterogeneous treatment effects over time. In *Robustness* below, we discuss this potential source of bias in greater detail, as well as the tests we conducted to ensure that it did not significantly drive our conclusions.

### Policy Implications.

Companies face growing pressure, both from their consumers and increasingly by the law, to rid their supply chains of MSL. In Brazil, major banks will soon require slaughterhouses to monitor both direct and indirect suppliers for socioenvironmental compliance in order to access credit. Beginning in July 2027, slaughterhouses that signed the TAC Cattle Agreements will be required to monitor indirect suppliers (15). Internationally, policies such as the California Transparency in Supply Chains Act and the UK Modern Slavery Act require corporations to report points in their supply chain where workers may be vulnerable. The EU recently moved to ban products made with MSL from their markets (16), and notably, the EU Deforestation Regulation will soon require importers of certain products—including cattle, beef, and leather—to conduct due diligence that all suppliers complied with country-of-origin labor laws and other legislation. To comply with these policies and fulfill commitments to remove labor infractions from supply chains, companies will require stringent traceability systems that allow visibility into upstream production practices.

As essential control points in the supply chain, slaughterhouses must adopt comprehensive traceability and monitoring systems that are difficult to evade. An important first step toward reducing the risk of laundering is the adoption of monitoring of indirect suppliers, which is already partially feasible with existing tools, particularly in states like Pará that have better data availability. Pressure from new requirements under the Beef TAC is accelerating the development of tools to aid with indirect monitoring, which will promote wider and more robust traceability in the sector. States like Pará and Mato Grosso are rolling out animal level traceability programs which may help reduce opportunities for laundering. MSL and environmental degradation often co-occur, presenting an opportunity for single systems to provide assurances related to both MSL and deforestation. These systems would reduce the monitoring burdens of slaughterhouses and downstream companies. If slaughterhouses fail to adopt comprehensive monitoring systems, downstream buyers may withdraw from the Brazilian cattle sector altogether (2, 16). Such a shift would open the door for new buyers from less discerning markets and could increase the prevalence of both MSL and deforestation.

Economic theory predicts that increasing the cost of being included on the Dirty List should increase the policy’s potential to deter MSL (see theoretical model and discussion in *SI Appendix*, section S4). In our sample, almost all Dirty Listed ranchers passed a labor inspection and were removed from the list after two years, and only four of 332 ranchers reappeared on the list after removal, suggesting that a combination of the costs of listing and increased scrutiny of perpetrators by law enforcement discouraged recidivism. Improved supply chain monitoring that includes indirect suppliers will increase deterrence potential by making evasion more costly and difficult, but expanded resources for government inspectors to identify and sanction more ranchers who use MSL is also needed. The level of resources designated toward labor law enforcement has fluctuated greatly during different political regimes, yet even during times of comparatively high levels of enforcement, the Dirty List is known to greatly underestimate the true extent of the problem (14, 17). Expropriation of assets of those found to have engaged in MSL, as is currently under consideration in the Brazilian legislature, would allow the government to seize cattle of implicated ranchers, thereby preventing them from entering supply chains and greatly increasing the costs of being listed (18). Increased pressure from both the public and private sector will be key to increasing the costs of engaging in MSL.

Overall, our results highlight the urgent need to strengthen the current strategies used to monitor and mitigate MSL in supply chains. With continued international pressure, private-sector action, and increased government support, the expansion of monitoring can ensure clean supply chains and reduce the economic incentives to employ forced labor.

## Materials and Methods

### Data.

#### Cattle transaction data.

Transaction data came from the Animal Transit Guide (Portuguese acronym GTA), a database that records livestock movements in Brazil for sanitary monitoring purposes (19–22). Federal law requires registration of paperwork prior to moving any animal to another ranch or slaughterhouse. Each record contains information on the date of transaction, number of head traded, sex and approximate age of the animals, selling and buying ranches, municipality, and purpose of the transaction. Each transaction includes seller and buyer identification numbers, which we use to match to identifiers on Dirty List records. The database is generally considered a comprehensive record of cattle movements in the country (9, 23).

#### Dirty list data.

Data on MSL came from Brazil’s Dirty List, which the Ministry of Labor is legally required to update every six months. Each entry on the list corresponds to a labor violation that the Ministry of Labor deemed to meet the legal definition of MSL, and includes information about the offending employer such as name, state, and identification numbers, as date of inclusion. We used the inclusion date to determine the first period in which each rancher was on the Dirty List and assumed a two-year duration of listing, consistent with official policy.^§^ The current list is publicly available, but the Ministry of Labor does not maintain a public repository of past lists (24). The Global Land Use and Environment Lab has obtained Dirty Lists since 2017 by downloading them directly from the Ministry of Labor website. Data on ranchers from older lists come from an Access to Information request that we submitted to the Brazilian government in April 2023.

In total, we were able to match 332 Dirty Listed ranchers to transaction records using information on the municipality and individual or firm identification numbers (CPF/CNPJ). *SI Appendix*, Fig. S1 breaks down when each of these ranchers were added to the list, showing that most ranchers were added between 2010 and 2013. The gap from 2014–2017 represents the outage time, during which the Dirty List was not publicized due to a court case challenging the constitutionality of the publicity of the list (*SI Appendix*, section S1). After the list came back online, there was a decline in the number of new ranchers added each year.

#### Transaction samples.

The sample included transactions from 2009 to 2023 from four states in the Brazilian Amazon. Exact temporal coverage varies by state due to changes in data access policies. Mato Grosso transactions span 2009–2018; Pará, 2013–2023; Rondônia, 2009–2017; and Tocantins, 2017–2023.

Our primary sample, the “Dirty List” or “DL” sample, included 168,984 transactions by 332 selling ranchers that were ever on the Dirty List and were matchable to the transaction records. As a robustness check, we estimated our models on a sample that included never-listed ranchers in the control group. We constructed this sample by conducting matching using spatial variables that were available only for a subset of ranchers (*SI Appendix*, section S2.1). This matched sample included 184,960 transactions by 275 Dirty Listed ranchers and 273 matched ranchers. *SI Appendix*, Table S1 summarizes the distribution of sample ranchers by state and whether the rancher appeared on the Dirty List during the study period. *SI Appendix*, Tables S2 and S3 present a breakdown of the transaction data by state in the Dirty List sample and matched sample.

To construct the rancher-level panel, we collapsed all outcome variables to the approximately six-month periods between when Dirty Lists were released. We weighted each outcome variable by the actual number of days in that period. Given the comprehensive nature of the transaction records (9, 23), when we created the panel we assumed that the absence of a transaction record for a given rancher and period meant that zero cattle were sold during that time. The panel only includes states and months for which there are transaction data available.

*SI Appendix*, Table S4 summarizes the means and SDs of the number of head sold by a rancher in a six-month period. We disaggregated by sales destination and sample (main, matched, or both). Ranchers who appeared on the Dirty List differed from never-listed ranchers in the matched sample in all outcome variables, with higher sale volumes to all types of buyers. However, the proportion of cattle that go to each type of buyer is relatively similar between ever- and never-listed ranchers; both groups sell more head indirectly than directly. Among direct sales, volumes are higher to slaughterhouses that signed the Cattle Agreements and lower to slaughterhouses that did not.

#### Cattle export data.

Our export data were obtained from PIERS, which compiles shipment-level records of international trade. We used data on exports of beef and leather from Brazil from the years 2014, 2018, and 2019 (25). These records include destination countries, as well as origin company and municipality name, which we used to link the export data to transactions with slaughterhouses from the transaction records database. We used the export data to determine whether slaughterhouses that were linked to Dirty Listed ranchers sold beef and leather products into international supply chains; we did not consider these data in the main econometric analysis.

#### Cost of cattle agreement sanctions data.

Ranchers blocked by Cattle-Agreement slaughterhouses can circumvent sanctions by selling cattle indirectly through compliant ranches (9, 12, 27). The transactions database (GTA) does not contain price information, so we were unable to observe whether ranchers faced price penalties for selling indirectly rather than directly. To derive approximate estimates of the costs associated with shifting to indirect sales after exclusion, we drew on a commissioned report produced by independent, locally based consultants, which provided aggregated estimates from April 2024 of selected transaction costs associated with indirect sales. These costs included i) transporting animals to a compliant (“clean”) ranch; ii) payments to intermediaries; iii) fees for issuing additional animal transit documents which may be needed to legitimize noncompliant sales. These estimates were based on information collected through interviews and market inquiries conducted by the consultants. We combined these estimates with data on cattle prices to contextualize the magnitude of these costs. *SI Appendix*, section S3 describes this analysis in greater detail.

#### Human subjects.

This study was conducted under University of Wisconsin–Madison Institutional Review Board protocol 2013-0044-CP021, which approved the use of the administrative data analyzed in this study under a waiver of informed consent. To inform the interpretation of certain cost parameters, we draw on a commissioned field report produced by independent, locally based consultants. For this, the authors received only aggregated, nonidentifiable estimates and did not access individual-level or identifiable data.

### Empirical Approach.

#### Staggered difference-in-differences model.

We used difference-in-differences analyses and an event study methodology to estimate changes in sales behavior after a ranch was added to and subsequently removed from the Dirty List. Similar to Chimeli and Soares, we allow for two distinct treatment periods (i.e., during and after listing) in a DiD framework (28). We estimated the following model:

[1] $$\begin{array}{c} SALE_{\mathrm{rbt}}=\beta_{0}+\beta_{1}OnDL_{\mathrm{rt}}+\beta_{2}FormerlyDL_{\mathrm{rt}}+\delta_{r}+\lambda_{t}+\epsilon_{\mathrm{rbt}}. \end{array}$$

The outcome of interest, $SALE_{\mathrm{rbt}}$, is the natural log of the number of head sold plus one for rancher *r* during a six-month period *t* to a given buyer type *b*. For a given six-month period, the dummy variable $OnDL_{\mathrm{rt}}$ equals one if the rancher was on the Dirty List and zero otherwise; the dummy variable $FormerlyDL_{\mathrm{rt}}$ equals one if the rancher was previously on the Dirty List and zero otherwise. We coded all transactions during the 2015–2017 Dirty List outage as unlisted or formerly listed (i.e. $OnDL_{\mathrm{rt}}=0$). Two-way fixed effects control for many unobserved factors that correlate with both appearing on the Dirty List and cattle transactions. Rancher fixed effects, $\delta_{r}$, control for time-invariant characteristics of individual ranchers in the sample, such as location or general operation size. Six-month-period fixed effects, $\lambda_{t}$, control for factors that vary across time but affect the entire sample, such as commodity prices and national-level policy changes. SEs, $\epsilon_{\mathrm{rbt}}$, are clustered at the rancher level.

We natural-log-transformed all outcome variables because semesterly sales were heavily skewed toward zero. In the main results table and figures, we present the raw estimated coefficients from this log–linear model. To contextualize the magnitudes of the coefficients, in *Results* narrative, we transformed the estimated coefficients and CIs to percent changes in sales by performing the transformation $(e^{\beta }-1)*100$. We also estimated our model with a Poisson pseudo-maximum likelihood (PPML) estimator for robustness (described below).

We estimated separate regressions for each type of sale destination. Direct transactions are those to slaughterhouses for the purpose of slaughter. For direct transactions, we estimated changes in sales to four different types of slaughterhouses: i) those that signed the PLC Cattle Agreement; ii) those that had signed TAC Cattle Agreement by the time of transaction (including those that had also signed PLC); iii) export-oriented slaughterhouses (i.e., those that are federally inspected) that had signed neither Cattle Agreement by the transaction time; and iv) non-export-oriented slaughterhouses (i.e., those that are state- or municipally inspected) that signed neither Cattle Agreement.

Indirect sales are transactions to other ranches for purposes of calving or fattening. We estimated changes in indirect sales of younger and older animals, defined as sales to other ranchers of animals younger and older than two years of age, respectively. We also estimated changes in indirect-PLC and indirect-TAC sales, defined as sales of animals over two years of age to ranches that subsequently sold animals to cattle agreement (PLC or TAC) slaughterhouses within one year of the indirect sale. These transactions were the most likely pathway into Cattle-Agreement supply chains, since they involved slaughter-age animals that were added to herds from which animals were sold directly to Cattle-Agreement slaughterhouses.

Our primary specification separately estimates the average difference in sales to various destinations during the two years a rancher appeared on the Dirty List and in the years after they were removed, relative to the years before listing. In the main text and Fig. 2, we present results from estimating these models with Ordinary Least Squares (OLS) and rancher and period fixed effects on the sample of ever-listed ranchers. Two-way fixed effects specifications are versatile, allowing for straightforward interpretation and flexible modeling of alternative specifications. This is particularly useful in our setting where treatment turns off but effects may persist.

Our average effects model does not differentiate effects by the number of years until or since Dirty List addition or removal. To assess how a rancher’s behavior evolved on a semesterly basis in the years before, during, and after listing, we also estimated an event study model with the de Chaisemartin and D’Haultfæuille estimator on the sample of ever-listed ranchers (29). This is our preferred event study estimator because it is robust to heterogeneous treatment effects over time and does not require never-treated units as controls. To ensure that our primary conclusions were not sensitive to our choice of sample, model, or estimator, we conducted a suite of complementary robustness checks, including estimation on an alternative sample, estimation of a PPML model, and estimation of the event studies with alternative estimators. We detail these analyses in *Robustness* below.

#### Causal identification.

Staggered DiD models estimate a treatment effect by exploiting variation in treatment timing. Our preferred specification follows a typical event study approach in which all units in the sample are eventually treated. This means that we compared transaction outcomes by ranchers who are treated (i.e. currently on the Dirty List) and those who were previously treated (i.e., were previously on the Dirty List) to those who will eventually go on it. It is also possible to estimate event study models with never-treated units as controls (as we do to verify the robustness of our main results), but we prefer the sample of only ever-listed ranchers for two main reasons.

First, this sample allows us to evaluate behavioral change by the types of ranchers that our policies target. The Dirty List and Cattle-Agreement sanctions aim to punish labor-exploiting ranchers, and all ranchers on the Dirty List perpetrated MSL. In contrast, the set of ranchers that were not added to the Dirty List may include some ranchers with MSL that were not caught, but most did not employ MSL. The policy response by ever-listed ranchers is thus more relevant than the potential response by an average rancher in our study states.

Second, the set of ranchers that were never added to the Dirty List does not necessarily strengthen (and in fact potentially weakens) our counterfactual. Dirty Listed ranchers had significantly different labor practices, productivity, and landholdings than the average never-listed rancher (*SI Appendix*, Table S4) (7). This means ever-listed and never-listed ranchers may not have responded in parallel to changes in economic, environmental, and political conditions over our study period, which could represent a threat to causal identification.

The main identification assumption for DiD models is parallel trends. In our study, this means that current Dirty Listed ranchers’ transaction behavior would have evolved in parallel to that of ranchers in the comparison group if they were not on the Dirty List at the time. Although this assumption is not directly testable, we evaluated its plausibility by assessing whether ranchers’ transaction behavior evolved in parallel to the comparison group in the two years before listing. For the outcomes of focus of this paper, the magnitude of estimated preperiod coefficients were small (roughly 5 to 20% relative to those during listing) (Fig. 3 and *SI Appendix*, Figs. S3–S11), suggesting that preexisting differential trends were unlikely to drive our findings. We discuss the identification assumption of no spillovers in *SI Appendix*, section S2.2.

### Robustness.

We conducted numerous tests to ensure that our findings were robust to alternative empirical specifications and that the Dirty List was the main driver of our results. Across specifications, we found that ranchers decreased direct sales to Cattle Agreement slaughterhouses while listed, and simultaneously increased indirect sales via clean ranchers to these same buyers. In this section, we describe the robustness tests we conducted. We discuss the results of these robustness tests in greater detail in *SI Appendix*, section S2.3.

#### Heterogeneity-robust event study estimation.

In staggered treatment settings, two-way fixed effects estimations may be biased under heterogeneous treatment effects due to the presence of negative weights on some cohort-time comparisons (29, 30). Unfortunately, there is no robust estimator that is flexible enough to match our main specification for estimating average effects. The method proposed de Chaisemartin and D’Haultfæuille estimates separate average effects of entry and exit from treatment, but identification requires that some units enter treatment simultaneously but exit at different times (29). In our setting, ranchers are listed for exactly two years. An earlier de Chaisemartin-D’Haultfæuille average effects estimator is robust to heterogeneous treatment effects and allows treatment to turn on and off. However, it relies on assumptions of symmetrical entry and exit into and out of treatment (i.e., the periods after treatment are a valid control equivalent to the periods before treatment), which our OLS and event study models show is likely violated for some outcomes (31).

To evaluate whether our conclusions were potentially biased due to negative weighting caused by heterogeneous treatment effects, we turn to our event study models, which we estimated with the heterogeneity-robust dynamic effects estimators from de Chaisemartin and D’Haultfæuille and Sun and Abraham (29, 32). We found that the sign, relative magnitudes, and statistical significance of the estimated coefficients on the event time dummies are similar to their corresponding during- and post-listing coefficients in the average effects model and consistent among event study specifications (*SI Appendix*, Figs. S3–S11), lending reassurance that negative weighting did not drive our results.

#### Alternative sample including never-listed ranchers.

We estimated our average effects model with OLS and two-way fixed effects on a matched sample that included never-treated ranchers (matching procedure described in *SI Appendix*, section S2.1). We conducted this check for additional robustness and to ensure comparability with the Sun-Abraham estimates, which relies on comparisons between ever- and never-treated units. For the average effects model, the matched sample results tended to closely match our primary specification (*SI Appendix*, Table S6). And for the event study model, the matched-sample results estimated with OLS and the Sun-Abraham estimator paralleled one another (*SI Appendix*, Figs. S3–S11).

#### Poisson model.

There are many rancher-period observations in our sample in which ranchers sold zero cattle to a given destination. In our primary specification, we addressed these zeroes by adding one to our outcome variables before natural-log transforming them. Nevertheless, zero inflation of the outcome variable may introduce bias and incorrect inference when using OLS. To check whether this bias was likely to have influenced our conclusions, we estimated our primary specification with a Poisson pseudo-maximum likelihood (PPML) model with rancher and period fixed effects (33). Our results—which are empirically similar to those from our primary specification and do not change any overarching conclusions—are presented in *SI Appendix*, Table S7.

#### Relationship between deforestation and dirty list timing.

Finally, we tested whether deforestation may be a confounding driver of our results. Administrative records indicate that ranchers often use MSL to clear forest, which represents an investment that enables the expansion of ranching activities as well as a potential reason why a slaughterhouse may block a rancher (7). To rule out the possibility that ranchers changed their sales behavior due to deforestation and not the Dirty List, we tested whether the timing of appearing on the Dirty List was correlated with the timing of deforestation (details in *SI Appendix*, section S2.3 and Table S8). We do not find a statistically significant increase in deforestation in the years preceding Dirty Listing, providing assurance that the Dirty List is the main driver of our results.

## Supplementary Material

Appendix 01 (PDF)

## Data Availability

Data on cattle movements come from the Guide to Animal Transport (GTA), which is a federal database tracking livestock movement. We are unable to share the GTA records as we have compiled them as this contains potentially sensitive information for individuals and violates the terms of our IRB. However, other researchers could also download individual GTA records and compile a similar dataset for study states for which the data is currently publicly available (19, 20). Records from past Dirty Lists came from an Access to Information request that we submitted to the Brazilian government. Similar to the GTA data, we are not able to share these lists, but other researchers could submit a request for this information or download contemporary lists from the Ministry of Labor (24). The export data that we used can be purchased from PIERS (25).

## Footnotes

^*^In Brazil, every slaughterhouse is either inspected by federal, state, or municipal authorities (“SIFs,” “SIEs,” and “SIMs,” respectively, from their Portuguese acronyms). SIFs are certified to sell products across state and country borders, while SIEs and SIMs tend to serve local markets. All Cattle Agreement slaughterhouses are SIFs, but not all SIFs signed the Cattle Agreements. In this paper, we refer to SIFs as export-oriented or export-certified slaughterhouses to differentiate them from SIEs or SIMs.

^†^All PLC slaughterhouses signed the agreement before our study period started and consequently the PLC implementation did not vary significantly by location or time during our study. In contrast, the TAC was gradually rolled out among different states and meatpacking companies throughout the course of our study (*SI Appendix*, Fig. S2). This means that we observe fewer ranchers who sold to TAC slaughterhouses before appearing on the Dirty List, reducing our statistical power to estimate differences in pre- and during-listing periods. Moreover, the novelty of the TAC during our study period perhaps contributed to greater variation in ranchers’ access to these slaughterhouses while on the Dirty List.

^‡^62% of indirect-PLC sales and 73% of indirect-TAC sales went to the same slaughterhouses to whom the Dirty Listed rancher sold while not on the list.

^§^Employers on the Dirty List can appeal their inclusion, which sometimes results in temporary or permanent removal from the public list. There is some evidence that politically connected employers are more likely to be removed early, but the average duration on the Dirty List is 1.93 y (26).
