# Supplementary material for: Laundering and evasion in response to antislavery policies in Brazil’s cattle sector
Source: Proc Natl Acad Sci U S A. 2026 Oct 12;123(42):e2532121123. doi: 10.1073/pnas.2532121123 (PMC13684292; doi:10.1073/pnas.2532121123)
Supplement: Supplementary file 1 [file pnas.2532121123.sapp.pdf]

PNAS

Supporting Information for
Laundering and Evasion in Response to Anti-Slavery Policies in Brazil's Cattle Sector

Tara Mittelberg
Marin E. Skidmore
Juliana L.S.C. Brandão
Lisa L. Rausch
Holly K. Gibbs

Corresponding authors: Tara Mittelberg, Holly K. Gibbs
Emails: mittelberg@wisc.edu, hkgibbs@wisc.edu

This PDF file includes:

- Supporting text
- Figures S1 to S11
- Tables S1 to S8
- SI References

Supporting Information Text

S1 Background

S1.1 Background on Modern Slave Labor (MSL) in Brazil

Globally, most individuals working in conditions of modern slave labor (MSL) work in the private sector, and 2.1 million are employed in agriculture [1]. MSL has multiple definitions, with most emphasizing the coercive means by which an employer extracts involuntary work. The International Labor Organization (ILO) uses the term forced labor as "all work or service which is exacted from any person under the threat of a penalty and for which the person has not offered himself or herself voluntarily" [2]. Therefore, determining whether someone is in MSL involves evaluating indicators of involuntariness (e.g., deceptive recruitment, poor working conditions, and long working hours) and coercion (e.g., threats of violence, exploitation of identity-related vulnerabilities, or debt bondage). The legal definition of MSL in Brazil encompasses a broader range of infractions than the ILO definition. Namely, it includes degrading working conditions, resulting in wider legal protections for workers.

Since 2004, Brazil's Ministry of Labor has published a semesterly "Dirty List" of employers that have been found to have subjected employees to MSL. Employers undergo a lengthy inspection and administrative process before appearing on the Dirty List. The process begins when the government or an NGO receives an anonymous tip of a labor violation. If the tip is deemed credible, a "Mobile Inspection Unit" conducts a labor inspection of the suspected firm. If the agents find evidence of MSL, they rescue the workers and refer the employer to the Ministry of Labor, which through an administrative procedure determines whether the working conditions did indeed constitute MSL. Employers deemed to have used MSL are ordered to pay fines and are added to the Dirty List [3].

The effective implementation of Brazil's Dirty List has faced significant obstacles due to political resistance from large employers and their allies. In December 2014, following pressure from an influential construction industry group, the president of the Supreme Court issued an injunction that resulted in the Dirty List being taken offline. It did not go online again until the Supreme Court overturned the injunction in March 2017. During the suspension period, the Ministry of Labor continued to internally update the names on the Dirty List but did not make them publicly available [4]. Additionally, there have been attempts to change or amend language defining MSL in the Penal Code. Employers argue that "degrading working conditions" is a vague term that allows normative interpretation by inspectors [5]. Political influences also contribute to fluctuations in enforcement. For example, during the Bolsonaro administration (2019-2022), the annual budget for combating MSL was 47% lower than that of preceding administrations [6]. Partially due to these political reasons, there was a significant decline in MSL enforcement after the Dirty List came back online in 2017 (Figure S1).

S1.2 The Brazilian Cattle Industry and MSL

Brazil is the largest producer of beef in the world, with cattle production making up 5% of the country's GDP [7]. Brazil is also the world's largest beef exporter, accounting for 19% of global exports in 2024, even with approximately 70% of its production being consumed domestically

[8]. In 2024, 44% of Brazil's cattle production took place in the nine states comprising the Amazon biome, where ranches are large and increasingly export-oriented [9, 8]. Most cattle are pasture-fed throughout their lifecycle, and ranchers often move animals between operations for calving and fattening. Rancher-slaughterhouse relationships tend towards spot-market purchasing, with only 10% of slaughter purchases negotiated using future contracts [10].

The nature of MSL in the Brazilian cattle industry has evolved in the decades since the Brazilian Penal Code first prohibited forced labor in 1945 and then debt bondage in 1965. In the second half of the twentieth century, government colonization policies in the Amazon, such as highway construction, facilitated land grabbing by wealthy producers [11]. Landowners relied on low-wage migrant workers who were isolated in large, sparsely settled areas and vulnerable to exploitation. Blatant violence, armed gunmen, and corruption were common under the authoritarian regime of the time [12]. Due in part to a greater threat of inspection by law enforcement and attention from NGOs in the last decades, labor coercion today is now less likely to be explicitly violent [12].

Instead, employers may deceive potential employees about the nature of the work to lure them to isolated rural cattle ranches. Once there, workers are subject to poor living quarters, unsafe working conditions, long hours, and little pay. Retention and manipulation of identity documents and wages are common means of control [13]. One recent analysis of Ministry of Labor inspection reports found that the most common MSL citations among cattle ranchers in Pará were for debt bondage and degrading working conditions, including inadequate provision of food, housing, toilets, or clean water [14]. Workers are often migrants from other municipalities, states, or countries and have low levels of education and literacy. These vulnerability factors reduce the likelihood of the worker recognizing they are being exploited and reporting their conditions to relevant authorities. Meanwhile, employers claim that such labor practices are necessary to achieve profits in a competitive global market where the Brazilian government imposes high taxes and strict environmental regulations [11].

S1.3 Zero-Deforestation Cattle Agreements and Cattle Laundering

The majority of large and export-oriented slaughterhouses in Brazil are signatories of zero-deforestation Cattle Agreements, which prohibit purchasing from Dirty Listed ranchers. These agreements arose after Greenpeace published a report in 2009 linking the Brazilian cattle industry to Amazon deforestation and the Federal Public Prosecutors began investigating and suing companies for buying from producers violating environmental and social laws. The four largest meatpackers in Brazil – Marfrig (purchased by Minerva in 2023), Minerva, JBS, and Bertin (later bought by JBS) – signed the "G4" agreement with Greenpeace, now called the Public Livestock Commitment (PLC). The PLC companies monitor suppliers in the Amazon biome for illegal deforestation. Due to data constraints at the time the agreement was signed, requirements to monitor deforestation in the Cerrado biome portion of the Legal Amazon (which makes up parts of Tocantins and Mato Grosso) were not included, but the Dirty List is monitored in these areas. Meanwhile, other meatpacking companies started signing legally binding "Terms of Adjustment of Conduct" (TACs) with federal prosecutors in each state. These "Beef TACs" require companies to address socio-environmental illegalities in their supply chains based on the legal concept of joint liability. The largest number of Beef TACs were signed in 2013 or 2014 (Fig. S1).

The majority of large and export-oriented slaughterhouses in Brazil are signatories of zero-deforestation Cattle Agreements, which prohibit purchasing from Dirty Listed ranchers. One of the greatest barriers to the success of the Cattle Agreements is the lack of traceability and control over indirect suppliers. Cattle typically live three years prior to slaughter and ranchers often move cattle between different properties for different stages of their lives such as breeding, rearing, and fattening. Despite the complexity of the supply chain, Cattle-Agreement slaughterhouses only monitor compliance with environmental and labor rules on the fattening farms that they buy from directly. Meanwhile, properties with illegal deforestation have been shown to sell to slaughterhouses that did not sign the Cattle Agreement or to launder cattle by selling to clean properties that eventually sell to PLC and TAC slaughterhouses [15, 16, 17, 18, 19, 20, 21]. Due to the public nature of the Dirty List, investigative journalists can and have found links between slaughterhouses and individuals on the list, which creates serious legitimacy risks for the implicated slaughterhouses [13].

Our study contributes to this literature in two key ways. First, it adds a more granular understanding of how ranchers respond to Cattle-Agreement sanctions, given our ability to isolate changes in outcomes at precise and frequent intervals by taking advantage of the availability of the listing and delisting dates. Notably, ranchers come off the Dirty List after two years, unlike sanctions for deforestation which are indefinite. This allows us to more credibly attribute any behavioral change to the sanctions rather than to other market forces. Second, we are the first to show systematic and widespread laundering to evade sanctions for MSL. Journalistic investigations and descriptive studies have documented cases of Dirty Listed ranchers selling cattle to other ranchers [13, 14, 22]. However, the data and statistical tools employed by these works do not permit conclusions about whether ranchers changed their behavior in response to being listed or unlisted. These studies are also limited in scope, either focusing on case studies or limited geographic areas. We use econometric methods and data from millions of cattle transactions across four states to show that laundering is significantly widespread and in direct response to loss of access to Cattle-Agreement slaughterhouses.

S2 Robustness

S2.1 Construction of the Matched Sample

For robustness and to allow for comparability between OLS and Sun-Abraham estimates (which rely on comparisons between ever- and never-treated units), we also used OLS to estimate our model on a matched sample. Ideally all non-Dirty List (control) ranchers would be similar to Dirty List (treated) ranchers, particularly in their labor practices and average output. However, we do not have data on labor practices, and we do not have sufficient pre-treatment data to match based on our outcomes.

Therefore, we estimated the models on a sample of listed ranchers and unlisted ranchers that share other observable attributes (see e.g., [23] and [24] for examples). We selected on physical attributes of the rancher's properties: the percent of forest remaining, the percent of the property in pasture, the percent of the properties in the Amazon and Cerrado biomes, and the straight-line distance to a federal highway. Both land use and location may be correlated with likelihood of MSL use: MSL is often used in remote areas and is employed in both deforestation and the cattle sector. We identify the set of unlisted matches using Mahalanobis

matching and one nearest neighbor.

Because we match ranches using attributes measured with spatial data, the sample of listed ranchers and candidate matches is limited to those whose properties that can be linked to a property registered in the Rural Environmental Registry (*Cadastro Ambiental Rural*). This excludes 18% of listed properties and may reduce the representativeness of the remaining sample [16]. We cannot match on sales characteristics entirely contained in the GTA, as all of these are potential outcomes of being listed. The matched sample also does not entirely remove level differences in outcomes between the listed and matched properties (Table S1). Because of these shortcomings, we believe that the set of assumptions required for identification using the set of listed properties is more likely to hold, and we view the matched sample as an informative secondary approach.

S2.2 SUTVA

For causal identification it is also necessary to make the Stable Unit Treatment Value Assumption (SUTVA), which states that the treatment states of one individual does not affect the outcomes of another individual in the sample (i.e. no spillover effects). A plausible violation of this assumption would be if a Dirty Listed rancher sold cattle to a non-listed neighbor and the neighbor then sold to Cattle-Agreement slaughterhouses. If pervasive enough, this violation would increase the magnitude of estimated coefficients. However, SUTVA is a reasonable assumption in the context of this dataset: In the Dirty List sample, the chance of spillover is negligible, since the 332 ever-listed ranchers are dispersed across four states with a total area of approximately 2.7 million square kilometers (roughly the size of Argentina).

S2.3 Discussion of Robustness Results

Here we discuss in more detail the results of three alternative models: event studies on the ever-listed sample using OLS, event studies and average effects models on a matched sample with never-listed using both OLS and heterogeneity-robust estimators, and a Poisson model. Though there are a few differences that we detail below, the overall similarity in our findings and conclusions reassure us that our overall conclusions are not influenced by bias due to our choice of estimator.

Heterogeneity-Robust Event Study Estimation

Results of the event studies using OLS on the sample of ever-listed ranchers are similar to the heterogeneity-robust estimates we report in the main text. We find a nearly identical pattern of decrease during listing and return to previous levels for direct sales to CA slaughterhouses (both TAC and PLC). We also find a similar increase during listing and return to previous levels for indirect sales to the PLC. However, we find a significant increase in indirect-TAC sales six months after listing using OLS, though the remaining coefficients during listing are still positive but insignificant. All coefficients during listing are positive but insignificant using our main heterogeneity-robust estimator. When we split indirect sales by animal age, we find identical results: indirect sales under 24 months were unchanged by listing, while sales over 24 months increased during listing and return to pre-listing levels after removal. Finally, we again find that

total sales, as well as direct sales to non-CA slaughterhouses, fell significantly in the second year of listing and did not fully recover after removal.

Alternative Sample Including Never-Listed Ranchers

Using a matched sample that includes never-listed ranchers allows us to make comparisons outside of listed ranchers and employ the heterogeneity-robust estimates of [25] (SA model). The conclusions are similar to those of the models using the ever-listed sample. We again find that direct-PLC and direct-TAC sales fell during listing, in a magnitude similar to the ever-listed sample. Notably, the estimates are much more precise using the SA model, possibly due to the larger sample size, which demonstrates with greater precision that, while direct-PLC sales increased once ranchers were removed from the Dirty List, they did not return to pre-listing levels. For indirect sales, we again find a significant increase in indirect sales followed by a return to pre-listing levels. Using the matched sample shows a more gradual return to pre-listing levels, including a still-significant positive coefficient for the first six months after removal using OLS on the matched sample. As before, the increase in indirect sales was entirely driven by sales of older animals (>24 months). The matched sample also confirms that listed ranchers decreased total sales in the second year that they were listed and did not fully recover, which is also reflected in a decrease in direct sales to non-CA slaughterhouses.

Poisson Model

The results of the Poisson model are similar to the log-linear model we report in the main text in terms of sign and significance with a few key differences. We find a significant decrease in direct-PLC and direct-TAC sales during listing that incompletely recovers after listing. Similarly, we find a smaller decrease in direct sales to non-CA slaughterhouses during and after listing that is similar in magnitude to the overall decrease in direct sales. One notable difference is that we do not find a significant increase in indirect sales to the PLC or TAC during listing compared to pre-listing levels, though the coefficients are positive and significantly different from post-listing sales behavior. We also continue to find a positive increase in overall indirect sales, and particularly for sales of animals over 24 months.

Relationship Between Deforestation and Dirty List Timing

It has been documented that some ranchers employ modern slave labor to clear forest for pasture [14]. Deforestation could confound our results if deforestation and not the Dirty List either caused ranchers to change their production practices or lose access to Cattle-Agreement slaughterhouses. To determine whether the timing of deforestation is systematically associated with the timing of appearing on the Dirty List, we implemented our OLS event study model to test for changes in deforestation (total hectares and percent of a rancher's holdings) in the years before and after a property is added to the Dirty List. Deforestation is published annually, so we structure the data annually relative to the year of listing. Since this analysis requires spatial information on the locations of ranchers' properties, we used the matched sample. We find no significant difference in deforestation on Dirty Listed properties in the years prior to the property being listed, either in terms of hectares of deforestation or as a percent of the total holding size (Table S8). We therefore conclude that Dirty Listed properties were not being blocked from slaughterhouses due to deforestation, but rather being on the Dirty List.

S3 Cost of Laundering Details

S3.1 Cost Data

Filing additional GTA paperwork

We obtained information on the cost of filing GTA paperwork from the relevant state sanitary agencies [26, 27, 28, 29].

Transporting animals

We derive estimates for the cost of transporting animals from a commissioned field report on cattle transaction costs and market practices produced by independent, locally based consultants. The authors of the report obtained quotes for truck rental rates from 20 freight companies in nine municipalities across all four of our study states in April 2024; specifically, trucks with the capacity of transporting 18 head of fattened steers (bois), or 20 head of fattened heifers (vacas) or breeding cows (novilhas). This is the most common size of truck available for rent in our study area, and is capable of traversing unpaved roads. Cost estimates included cost of gas and a driver. The report included quotes for the cost-per-head of transporting animals distances of 21 and 100 kilometers, which correspond to the 50th and 80th percentile distances of indirect sales in our study area [30].

Fee to intermediary rancher

It was not possible to directly observe the fees that compliant ranchers may have charged Dirty Listed ranchers for facilitating indirect sales to Cattle Agreement slaughterhouses. To approximate a plausible range, we instead averaged the costs of other common intermediation services in the sector, such as sourcing or transporting calves or unfinished cattle. These estimates are drawn from the commissioned field report described in the previous paragraph, which compiles aggregated cost data for intermediation services based on interviews and market inquiries conducted across nine municipalities in the study region in April 2024.

Cattle price and revenue data

We normalized our cattle price and revenue data across multiple sources to estimate the cost of laundering. Some data that informed our cost estimates were reported per arroba (the 15 kg unit of weight used to measure finished animals in Brazil), while others were reported per animal or as percentages of revenue. To allow for comparison across costs, we converted all costs to cost per fattened steer and fattened heifer. We assumed that a fattened steer weighs 20 arroba and a fattened heifer weighs 15 arroba [31]. We calculated costs as a proportion of expected revenue per animal using average price data for fattened steers and heifers from our study states from May 2023-April 2024 [32]. We have state-level data for most costs of laundering. To calculate average overall expected revenue loss, we weighted state-level cost data by the number of Dirty Listed ranchers in our sample from each state (Table S1).

S3.2 Results

Despite being blocked from direct sales by Cattle-Agreement slaughterhouses, Dirty Listed ranchers maintained access to these same slaughterhouses by supplying them indirectly. This raises the question of the extent to which Cattle-Agreement sanctions impose an economic cost to ranchers, an important consideration for whether such sanctions may ultimately serve to deter MSL (Section S4).

Our econometric results suggest that the average Dirty Listed rancher faces at least some cost: After being removed from the Dirty List, ranchers switch back to supplying Cattle-Agreement slaughterhouses directly (Fig. 2), implying that this is their preferred transaction mode. There are three main economic penalties potentially faced by a sanctioned rancher not able to sell directly to a Cattle-Agreement slaughterhouse: 1) filing additional GTA paperwork; 2) transporting animals to a clean ranch; 3) paying a middleman. A given Dirty Listed rancher may face one or more of these costs as they circumvent Cattle-Agreement sanctions.

Filing GTA Paperwork

A Dirty Listed rancher who sold cattle indirectly to a Cattle-Agreement slaughterhouse would, at a minimum, have to file an additional GTA form so the cattle were under the name of a clean rancher who would then sell to the slaughterhouse. The cost per animal of filing this paperwork is R$4.72 in Mato Grosso, R$1.40 in Tocantins, R$4.58 in Pará, and R$4.43 in Rondônia. This is a trivial expense given that in 2023-2024, the average rancher in our study states could expect to make R$4143 per fattened steer and R$2843 per fattened heifer if they sold directly to a slaughterhouse. In some cases, filing an extra GTA form would be the only additional cost that a Dirty Listed rancher would face compared to a rancher who is able to sell directly to a Cattle-Agreement slaughterhouse. In other cases, a rancher may have to transport animals to a clean ranch and/or pay a cut to the rancher who intermediated the sale.

Transportation costs

The median and 80th percentile distances for indirect sales in our study area are 21 km and 100 km [30]. The estimated cost of hiring a truck to move an animal 21 km is R$29 for a fattened steer and R$26 for a fattened heifer. To move an animal 100 km costs R$59 per fattened steer and R$53 per fattened heifer. Aggregating across total sales a rancher could expect to make over two years on the Dirty List, moving animals 21 km would cost the rancher 0.8% of the potential revenue from selling directly to a Cattle-Agreement slaughterhouse, while moving animals 100 km represents a 1.6% cost. These percentages are very low, and are likely an upper bound, given that they represent quotes from freight companies and not final negotiated contracts paid by ranchers.

Payments for transportation are not always necessary. In some cases, a Dirty Listed ranch may be located close enough to a clean ranch that the rancher wouldn't need a truck to move animals between them. The median distance for an indirect sale in our study area is just 21 km, though Dirty Listed ranches tend to be larger, and thus likely farther from neighbors, than the average ranch [30, 14]. In other cases, sanctioned ranchers do not physically move animals to the clean ranch under which the new GTA paperwork is filed. When a rancher wishes to sell cattle for slaughter, the meatpacking company typically contracts a freight operator to transport the animals from ranch to slaughterhouse. The freight operator arranges with the

rancher to pick up the animals and does not necessarily check whether the ranch from which they pick up cattle is the same as the ranch detailed in the GTA paperwork for the transaction. Slaughterhouses only verify that the ranch named in the GTA paperwork is compliant with environmental and labor regulations. In other words, sanctioned ranchers can sell cattle from their own property and simply file the GTA paperwork under someone else's name.

Fee to intermediary rancher

While it's likely that not all middlemen charged Dirty Listed ranchers a fee for laundering cattle, in some cases it's plausible that they did. Slaughterhouses do not check the transaction history of their direct suppliers, and supply chain agreements do not currently explicitly penalize ranchers who purchase cattle from other non-compliant ranchers. Middlemen thus do not currently face direct economic costs from Cattle-Agreement buyers for laundering cattle. It's therefore likely that some middlemen did not charge a fee for selling cattle for sanctioned ranchers. Others may have charged a fee. They may face non-economic costs such as inconvenience or moral discomfort, or costs associated with holding the sanctioned rancher's animals on their property before sale to slaughterhouses. Moreover, it's common for other types of intermediaries in our study area to charge for their services, offering clues for the magnitude of fees that would be reasonable to charge.

In our study area, intermediaries known as *catireiros* usually mediate sales of unfinished cattle. Some ranchers contract an intermediary to source cattle for them, usually at earlier stages of the animal's life. The rancher then raises, reproduces, and/or eventually fattens these cattle for slaughter. Ranchers typically pay these intermediaries a commission of approximately R$20-R$30 per head, which represents approximately 1% of the value of the animal and covers the costs of sourcing the animal, evaluating its quality, and negotiating the sale. Transport costs are an additional expense typically incurred by the buying rancher. Another type of intermediary aggregates calves or unfinished animals from smaller ranches and transports them to larger ranches for backgrounding or finishing. These buyers typically charge a commission of R$50-R$150 per head, representing approximately 3-6% of the value of the animal. This fee typically includes transportation. In these transactions, ranchers tend to benefit from scale, with fees per animal decreasing as the number of animals sold increases.

Unlike intermediaries who help sanctioned ranchers launder finished cattle to Cattle-Agreement slaughterhouses, most *catireiros* facilitate sales of unfinished animals between unsanctioned ranchers. However, their usual commission, 1-6%, helps benchmark a going rate of simple intermediation with and without transportation included. The report also provides qualitative evidence that some firms advertise intermediation services to producers seeking to access downstream buyers despite market restrictions. At first these companies simply "loaned" GTA paperwork to sanctioned ranchers, but later directly purchased and transported animals from sanctioned ranches and then resold them for slaughter. These intermediaries reportedly took revenue cuts amounting to approximately 8-14% of the value of the animal.

S4 Theoretical Model

We model how the Dirty List and supply chain exclusion policies influence a rancher's decision to use MSL. Our model combined with our empirical estimations of the policies' effects on

cattle sales provide intuition for how these policies may be working (or not) to deter MSL.

We consider the Dirty List and Cattle Agreements in the context of the broader literature on crime and deterrence. According to Becker [33], the decision to commit a criminal offense is a function of the probability of conviction (ρ), the punishment per offense if convicted (f), and a bundle of other attributes including but not limited to the income available in legal and other illegal activities and the willingness to commit an illegal act (U). He models the supply of offenses by an individual as $O = O(\rho, f, U)$. The expected utility of an offense for a perpetrator is:

$$EU = \rho U(Y - f) + (1 - \rho)U(Y). \tag{1}$$

Becker captures the entire income from an offense in the term Y. We assume that a rancher's utility is increasing in income such that $\frac{\partial U}{\partial Y} > 0$.

We model income from cattle, Y, as:

$$Y = pQ(l) - tl. \tag{2}$$

Ranchers produce cattle according to production function $Q(l)$, where labor, l, is the only input. While a typical rancher would produce by paying wage w, we consider a rancher that employs MSL. The rancher no longer pays wages, but instead pays a per-unit labor cost $t < w$ for sourcing and maintaining forced laborers.

We consider the rancher that is choosing whether to employ MSL. If the rancher chooses to use MSL (i.e. $O >$ 0), they will pay wages t with certainty and be placed on the Dirty List with probability $0 \leq \rho \leq 1$. If they are placed on the Dirty List, they face a supply chain exclusion penalty (i.e. Cattle-Agreement sanctions), β, which we model as a penalty on the price p the rancher receives per animal sold [34]. The penalty is a general term that encompasses lower prices at non-monitoring slaughterhouses or the transaction and transportation costs associated with selling indirectly. Dirty Listed ranchers also incur costs f that are unrelated to their production. This term may include fines, legal fees, or sanctions from the banking industry.

Combining equations 1 and 2, the rancher's expected utility from committing an act of MSL is thus:

$$EU = \rho U((1 - \beta)pQ(l) - tl - f) + (1 - \rho)U(pQ(l) - tl). \tag{3}$$

We assume that a rancher's choice of O increases as the expected utility of a MSL offense increases. We can thus consider the impact of β on the rancher's choice[1] of O with the first order condition:

$$\frac{\partial EU}{\partial \beta} = \rho \frac{\partial U}{\partial Y}\left((-pQ(l) + (1 - \beta)p\frac{\partial Q}{\partial l}\frac{\partial l}{\partial \beta} - t\frac{\partial l}{\partial \beta}\right) \tag{4}$$

The first term, $\rho\frac{\partial U}{\partial Y}$, is strictly positive. It captures the probability of being added to the list times the marginal utility of income.

[1]We model a rancher's choice of employing MSL as continuous rather than binary because there are a range of offenses that constitute MSL. Government fines (captured in f), probability of being caught (ρ), and presumably income (Y) are increasing based on the severity of an offense.

We can decompose the second term, $(-pQ(l)+(1-\beta)p\frac{\partial Q}{\partial l}\frac{\partial l}{\partial \beta}-t\frac{\partial l}{\partial \beta})$ into (a) the direct loss in profit due to the price penalty of β on each unit sold, (b) the indirect loss in profit due to the lower choice of labor l and subsequent loss in quantity produced, and (c) the profit increase given the lower input cost due to the reduction in l.

A profit-maximizing rancher on the Dirty List will choose labor such that $\frac{\partial Q}{\partial l} = \frac{t}{(1-\beta)p}$. We can substitute this and find:

$$\frac{\partial EU}{\partial \beta} = \rho \frac{\partial U}{\partial Y}\Bigg((-pQ(l) + (1-\beta)p\frac{t}{(1-\beta)p}\frac{\partial l}{\partial \beta} - t\frac{\partial l}{\partial \beta}\Bigg) \tag{5}$$

Thus, the lost marginal revenue due to the lower choice of l is exactly balanced by the foregone input cost, and subsequently:

$$\frac{\partial EU}{\partial \beta} = \rho \frac{\partial U}{\partial Y}\Bigg(- pQ(l)\Bigg) \tag{6}$$

The lost profit is driven by the direct impact of the price penalty on profit. This term is negative, and we find that $\frac{\partial EU}{\partial \beta} < 0$. This leads us to our main result from the model:

> **Claim 1:** A higher market exclusion penalty β reduces the expected payoff of a MSL offense, and thus serves as a deterrent for MSL.

The size of the fines, f, and the probability of sanction, ρ, are also critical parameters for a rancher's optimal choice of O. As in Becker, $\frac{\partial O}{\partial \rho} < 0$ and $\frac{\partial O}{\partial f} < 0$. Two results follow:

> **Claim 2:** A higher probability of inspection, ρ, decreases the expected payoff of a MSL offense. Therefore ranchers are less likely to commit MSL in an environment with a high risk of being caught and sanctioned.

> **Claim 3:** Higher fines imposed by the government, f, reduce the expected payoff of a MSL offense and serve as a deterrent for MSL.

There are thus three main levers for reducing the expected payoffs of using MSL. Supply chain exclusion is a measure outside of the legal system that deters labor exploitation by imposing an additional loss in income conditional on being caught. Alternatively, the government can increase the probability of being caught or increase the cost of being caught.[2]

Notably, ranchers that have already been found to have MSL and are on the Dirty List are subject to increased scrutiny, including labor inspections by law enforcement. This means that these ranchers represent a special case for whom the probability of being caught, ρ, is effectively one. Substituting $\rho = 1$ into Equation 6, we can see that relative to other ranchers (for whom $0 \leq \rho < 1$), Dirty Listed ranchers' expected utility from re-committing a MSL offense decreases the most as price penalties increase. This implies that high price penalties can also encourage ranchers to return to clean production.

[2]Per Becker, the optimal choice of ρ and f balances the marginal damage to society from the crime, the marginal cost of enforcement and punishment, and the cost to society of punishment (e.g., the losses to families of people being punished and the costs for those employed in the carceral system.)

S4.1 Relationship with Empirical Findings

Our theoretical model helps connect our empirical findings with the policy's potential impact on MSL. Our model predicts that a stricter price penalty, β, serves as a stronger deterrent for ranchers considering employing MSL as well as a motivation for those on the Dirty List to return to clean production.

We can imagine a scenario where $\beta = 1$ represents a fully binding market exclusion policy. In this case, there is full adoption of monitoring by all buyers in the market and full supply chain monitoring transparency, so no indirect sale is possible. The rancher has no ability to sell their product and earns zero profit. While this policy has not been achieved in any supply chain of which we are aware, it is a useful exercise to consider this as the comparison case.

Our empirical results show that Dirty Listed ranchers were able to offset some of their losses in direct sales to Cattle-Agreement slaughterhouses by supplying non-monitoring slaughterhouses or Cattle-Agreement slaughterhouses indirectly. For ranchers able to launder finished animals, the price penalty for doing so was low – approximately 0.1-14% of expected revenue from selling directly to a Cattle-Agreement slaughterhouse. Therefore, our empirical results can be interpreted as a reduction in the value of β from 1 to 0.001-0.14, and a weakening of the deterrence effect of the policy.

In our model, and in the real context that we study, the supply chain policy exists within a larger legal and policy environment. As we model, there are additional costs, f, associated with being on the list apart from those derived from the Cattle Agreements. For example, firms convicted of MSL also pay government fines proportionate to the severity of their offense. In extreme cases these fines can exceed one million dollars [35]. We are unable to disentangle the impacts of the threat of continued exclusion from the supply chain from these additional costs. However, the additional costs outside of the Cattle Agreements may help to explain why Dirty Listed ranchers return to compliant production within two years, even though laundering lessens the severity of the price penalty, β.

Observing the Dirty List through the lens of our theoretical model also highlights the importance of ρ, the rancher's belief they will be caught, as a deterrent to employing MSL. The combination of policies that currently exist in Brazil has not eradicated MSL from all supply chains, as evidenced by continued violations. However, the subset of ranchers that know with certainty that they will be inspected and will face further punishment if they employ MSL all return to compliance. It appears that the combination of policies and punishments are severe enough to induce change, but the low risk of being listed reduces their threat sufficiently for MSL to continue.

Supplemental Figures

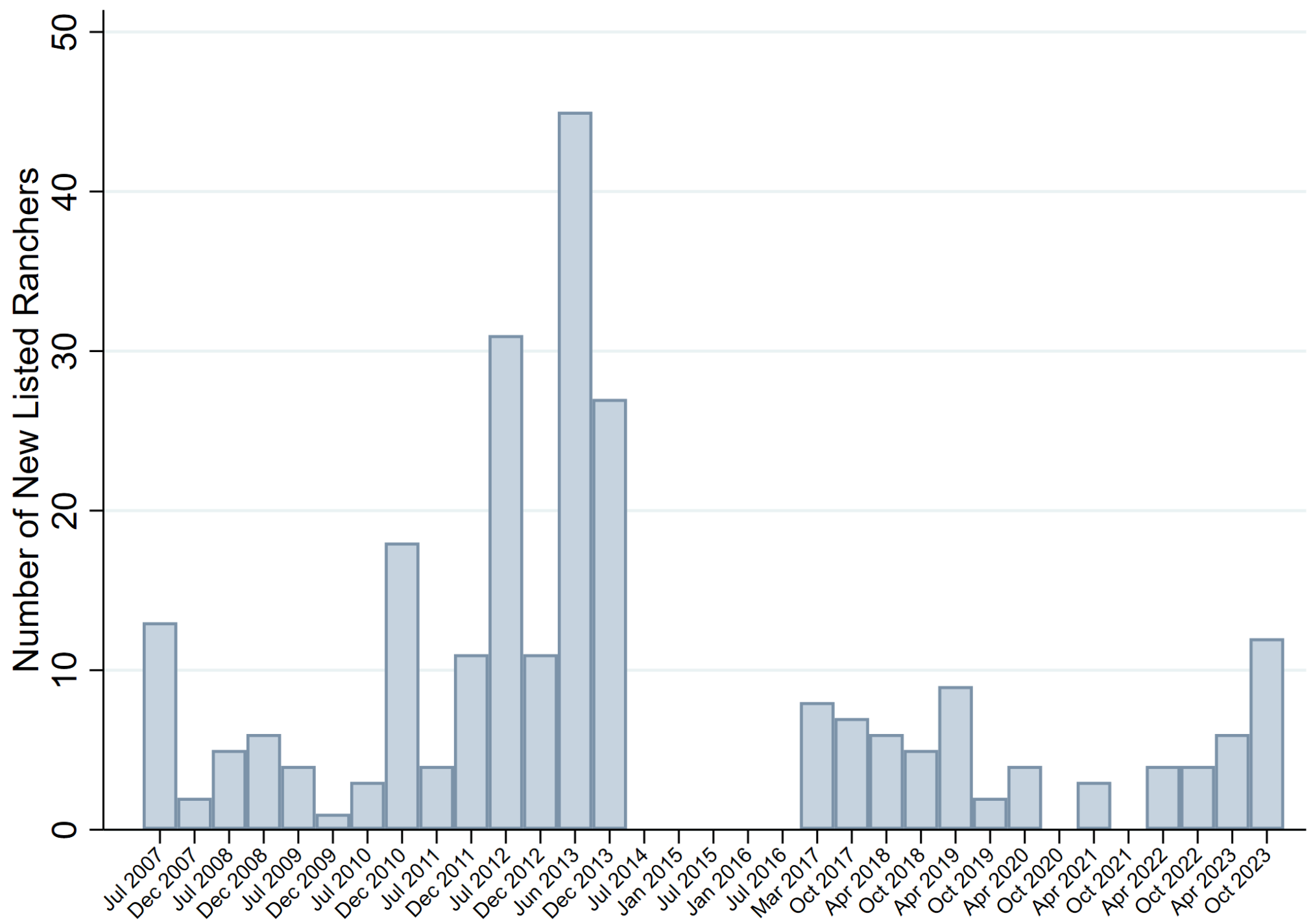

Figure S1: Number of new ranchers added to each Dirty List

The Dirty List was not publicized from 2014-2017. See details in Section S1.1.

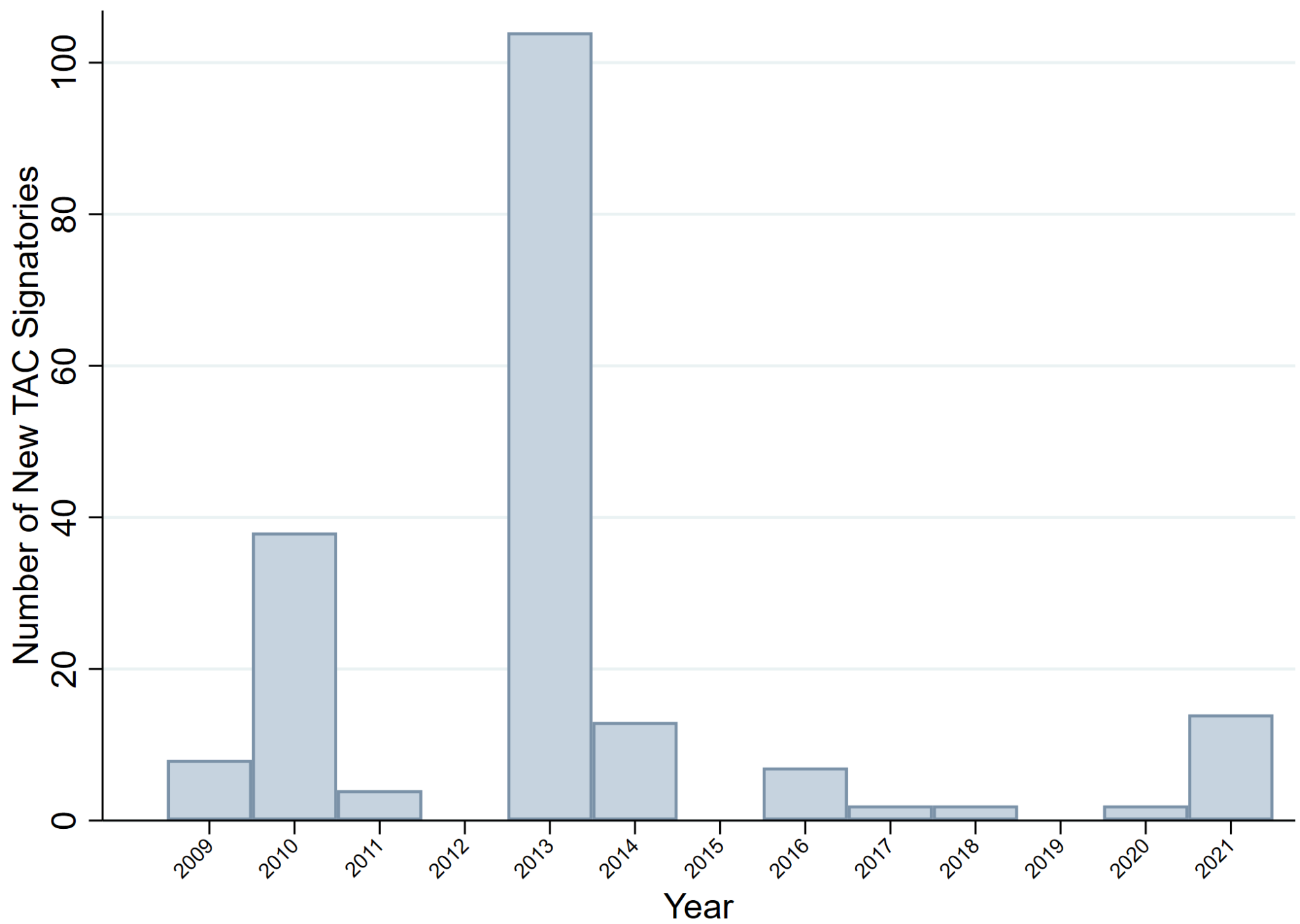

Figure S2: Number of new slaughterhouses in sample joining the TAC Cattle Agreement

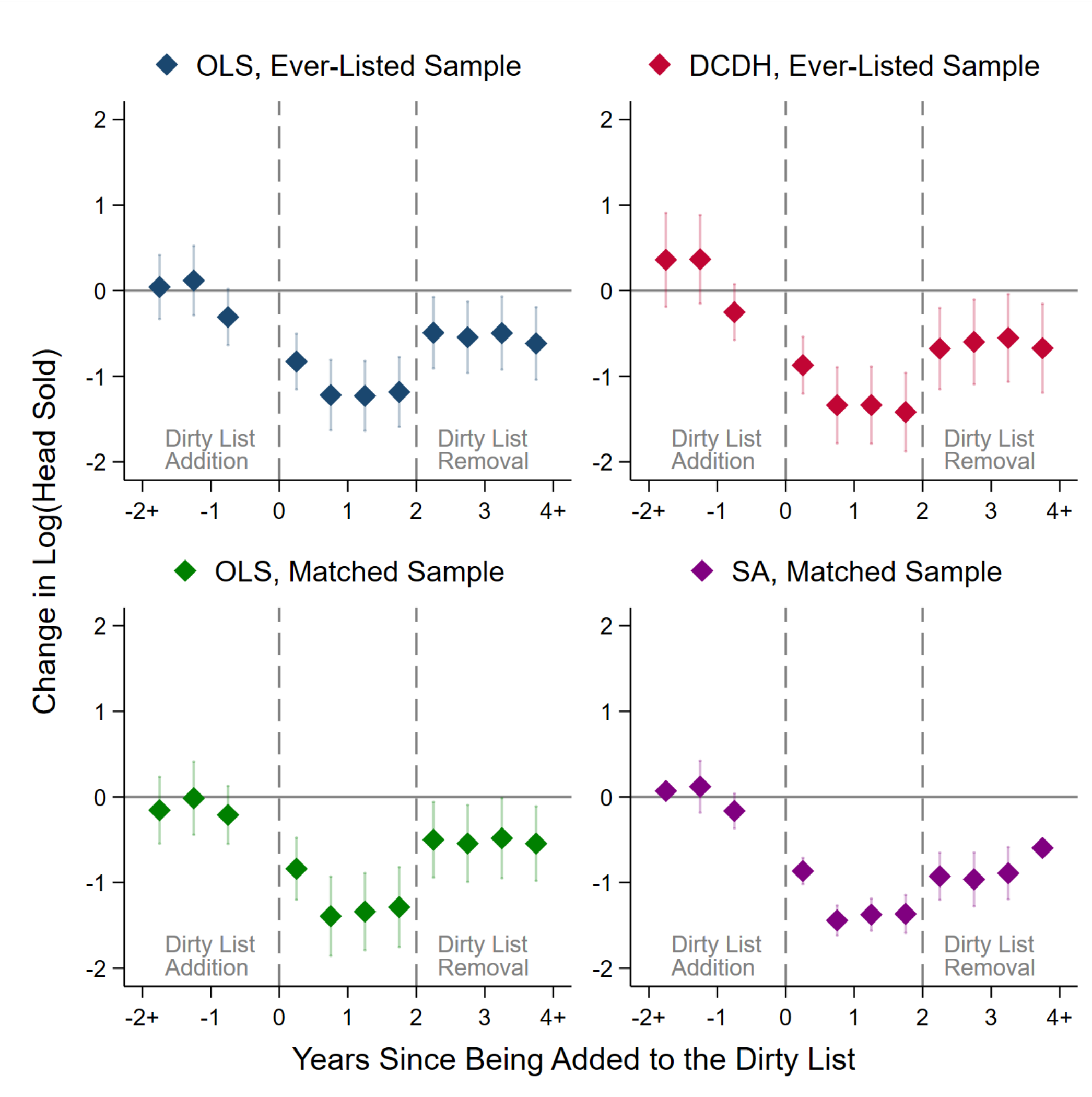

Figure S3: Changes in Direct-PLC sales over time

Bars represent 95% confidence intervals. Reference period is one semester prior to Dirty List addition. Results presented from testing changes in direct sales to PLC Cattle Agreement slaughterhouses over time using four different specifications: OLS with rancher and period fixed effects on the sample of ever-Dirty-Listed ranchers (robustness specification); the de Chaisemartin-D'Haultfœuille (DCDH) estimator on the sample of ever-Dirty-Listed ranchers (preferred specification); OLS with rancher and period fixed effects on the "matched sample" consisting of a subset of ever-listed ranchers plus matched never-listed ranchers (robustness specification); and the Sun-Abraham (SA) estimator on the matched sample (robustness specification).

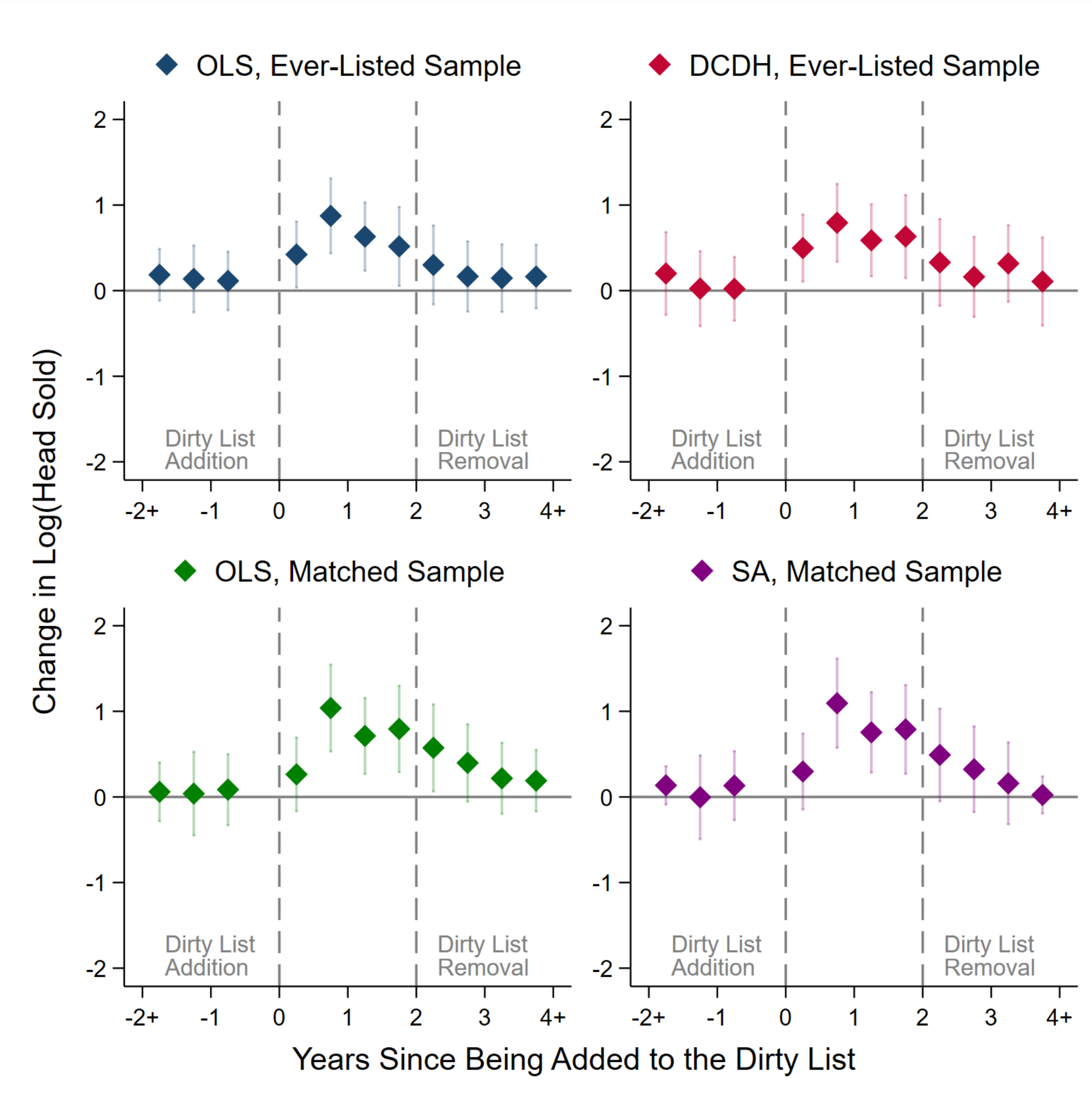

Figure S4: Changes in Indirect-PLC sales over time

Bars represent 95% confidence intervals. Reference period is one semester prior to Dirty List addition. Results presented from testing changes in sales of slaughter-age animals to other ranchers who went on to sell to PLC Cattle Agreement slaughterhouses within one year (“Indirect-PLC” sales) using four different specifications: OLS with rancher and period fixed effects on the sample of ever-Dirty-Listed ranchers (robustness specification); the de Chaisemartin-D’Haultfœuille (DCDH) estimator on the sample of ever-Dirty-Listed ranchers (preferred specification); OLS with rancher and period fixed effects on the “matched sample” consisting of a subset of ever-listed ranchers plus matched never-listed ranchers (robustness specification); and the Sun-Abraham (SA) estimator on the matched sample (robustness specification).

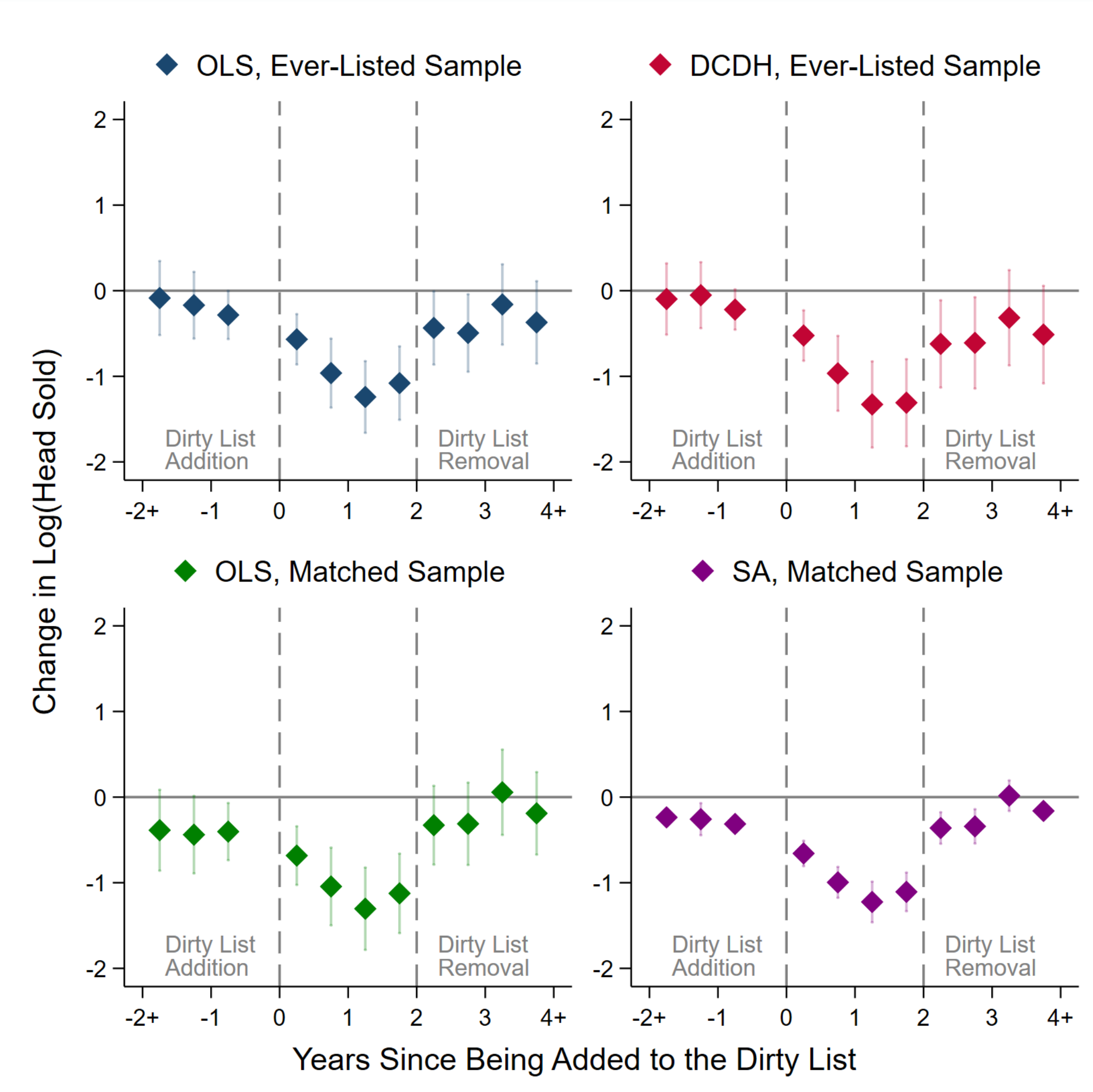

Figure S5: Changes in Direct-TAC sales over time

Bars represent 95% confidence intervals. Reference period is one semester prior to Dirty List addition. Results presented from testing changes in direct sales to TAC Cattle Agreement slaughterhouses over time using four different specifications: OLS with rancher and period fixed effects on the sample of ever-Dirty-Listed ranchers (robustness specification); the de Chaisemartin-D'Haultfœuille (DCDH) estimator on the sample of ever-Dirty-Listed ranchers (preferred specification); OLS with rancher and period fixed effects on the "matched sample" consisting of a subset of ever-listed ranchers plus matched never-listed ranchers (robustness specification); and the Sun-Abraham (SA) estimator on the matched sample (robustness specification).

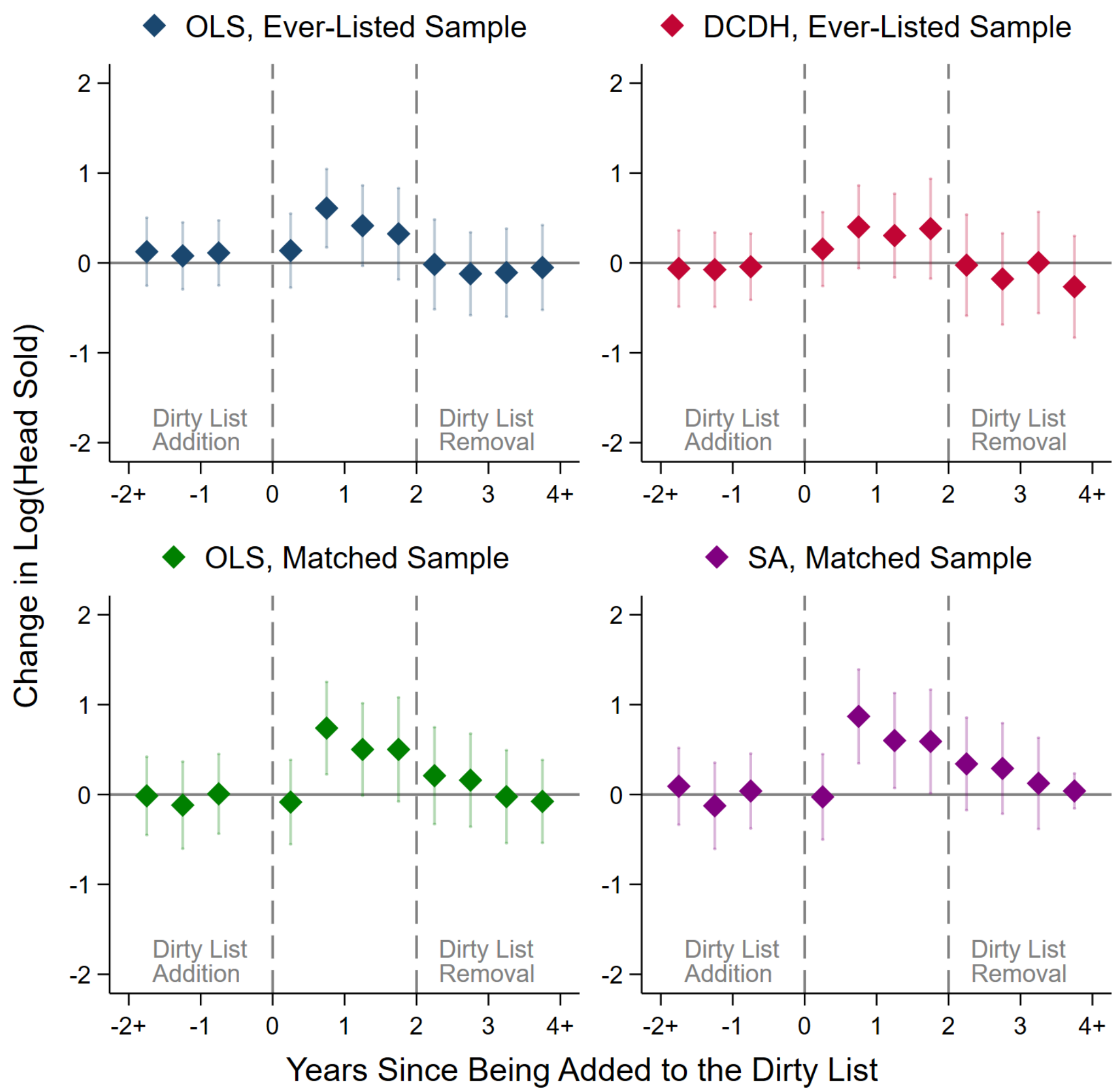

Figure S6: Changes in Indirect-TAC sales over time

Bars represent 95% confidence intervals. Reference period is one semester prior to Dirty List addition. Results presented from testing changes in sales of slaughter-age animals to other ranchers who went on to sell to TAC Cattle Agreement slaughterhouses within one year ("Indirect-TAC" sales) using four different specifications: OLS with rancher and period fixed effects on the sample of ever-Dirty-Listed ranchers (robustness specification); the de Chaisemartin-D'Haultfœuille (DCDH) estimator on the sample of ever-Dirty-Listed ranchers (preferred specification); OLS with rancher and period fixed effects on the "matched sample" consisting of a subset of ever-listed ranchers plus matched never-listed ranchers (robustness specification); and the Sun-Abraham (SA) estimator on the matched sample (robustness specification).

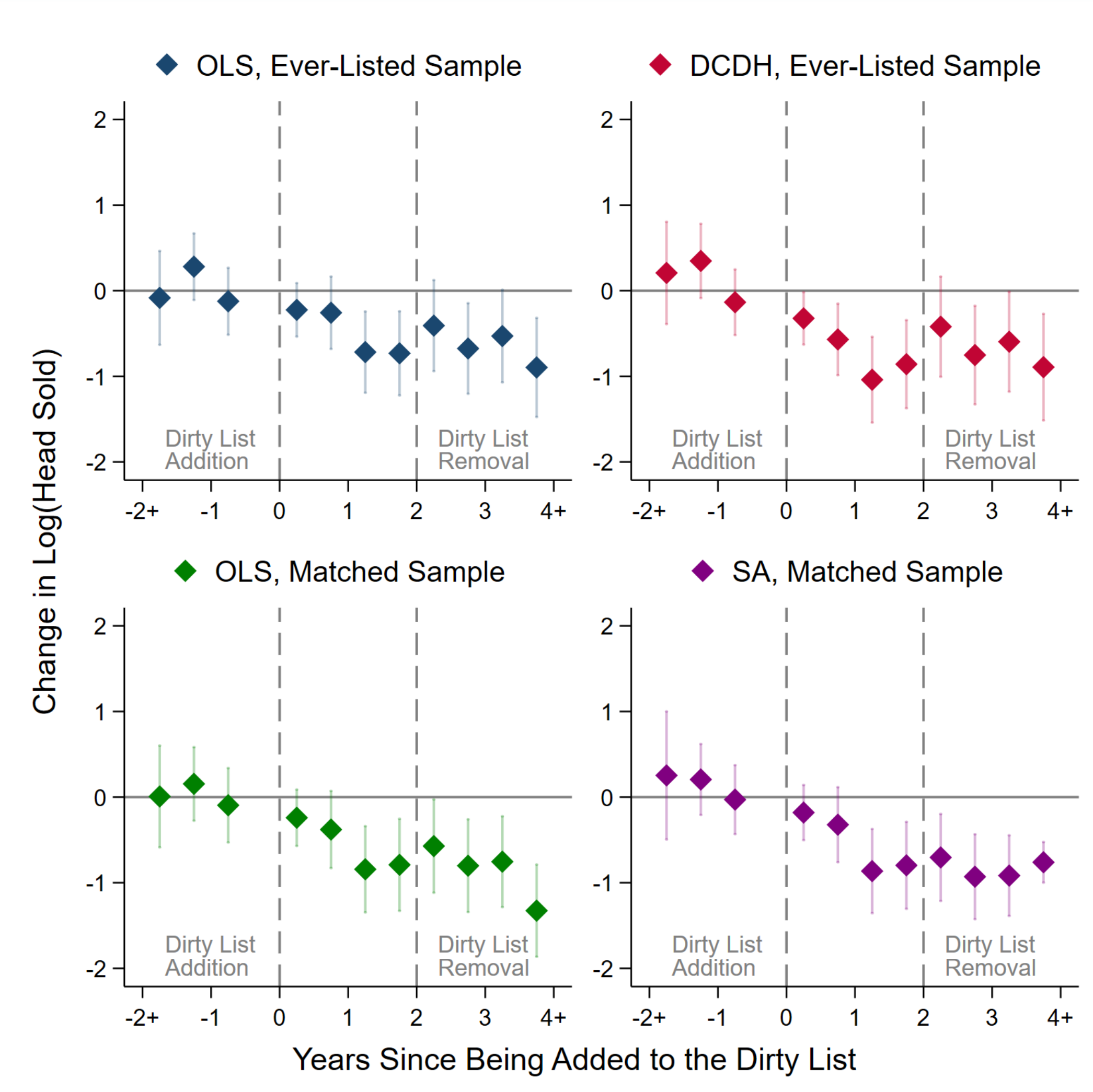

Figure S7: Changes in all sales over time

Bars represent 95% confidence intervals. Reference period is one semester prior to Dirty List addition. Results presented from testing changes in all sales using four different specifications: OLS with rancher and period fixed effects on the sample of ever-Dirty-Listed ranchers (robustness specification); the de Chaisemartin-D'Haultfœuille (DCDH) estimator on the sample of ever-Dirty-Listed ranchers (preferred specification); OLS with rancher and period fixed effects on the "matched sample" consisting of a subset of ever-listed ranchers plus matched never-listed ranchers (robustness specification); and the Sun-Abraham (SA) estimator on the matched sample (robustness specification).

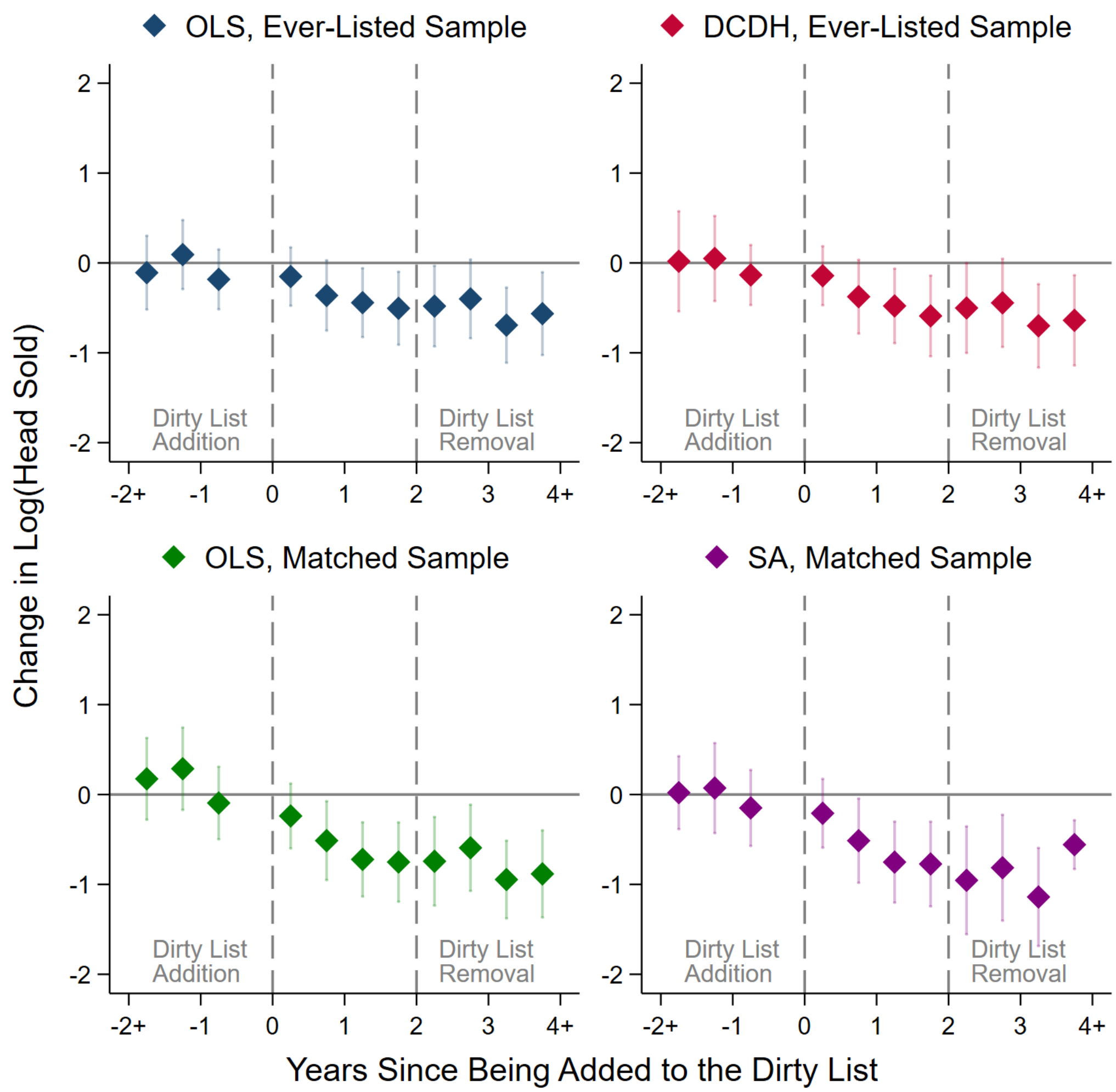

Figure S8: Changes in sales to export-certified non-Cattle-Agreement slaughterhouses over time

Bars represent 95% confidence intervals. Reference period is one semester prior to Dirty List addition. Results presented from testing changes in direct sales to export-certified non-Cattle-Agreement slaughterhouses (SIFs) over time using four different specifications: OLS with rancher and period fixed effects on the sample of ever-Dirty-Listed ranchers (robustness specification); the de Chaisemartin-D'Haultfœuille (DCDH) estimator on the sample of ever-Dirty-Listed ranchers (preferred specification); OLS with rancher and period fixed effects on the "matched sample" consisting of a subset of ever-listed ranchers plus matched never-listed ranchers (robustness specification); and the Sun-Abraham (SA) estimator on the matched sample (robustness specification).

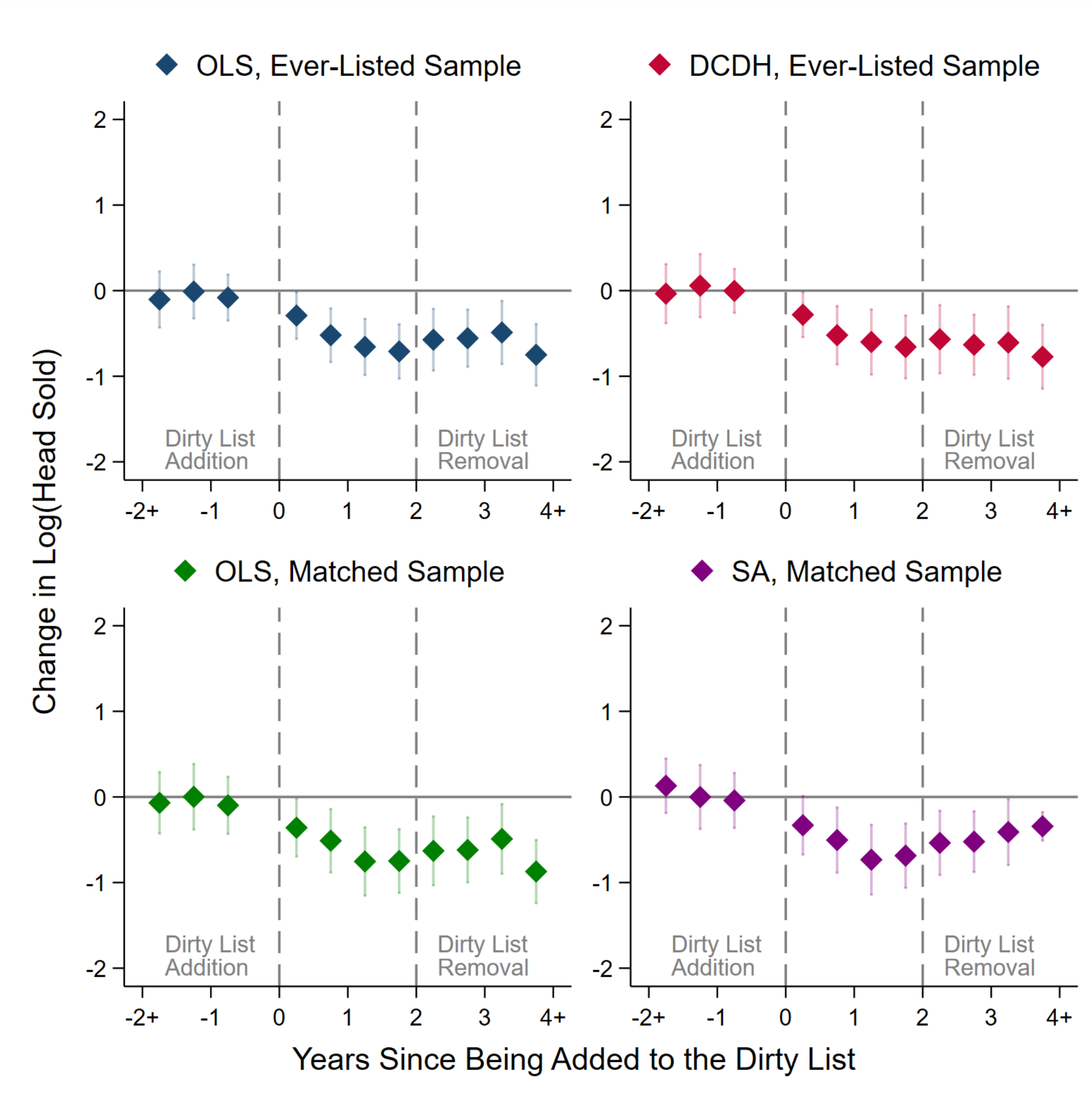

Figure S9: Changes in sales to non-export-certified non-Cattle-Agreement slaughterhouses over time

Bars represent 95% confidence intervals. Reference period is one semester prior to Dirty List addition. Results presented from testing changes in direct sales to non-export-certified non-Cattle-Agreement slaughterhouses (non-SIFs) over time using four different specifications: OLS with rancher and period fixed effects on the sample of ever-Dirty-Listed ranchers (robustness specification); the de Chaisemartin-D'Haultfœuille (DCDH) estimator on the sample of ever-Dirty-Listed ranchers (preferred specification); OLS with rancher and period fixed effects on the "matched sample" consisting of a subset of ever-listed ranchers plus matched never-listed ranchers (robustness specification); and the Sun-Abraham (SA) estimator on the matched sample (robustness specification).

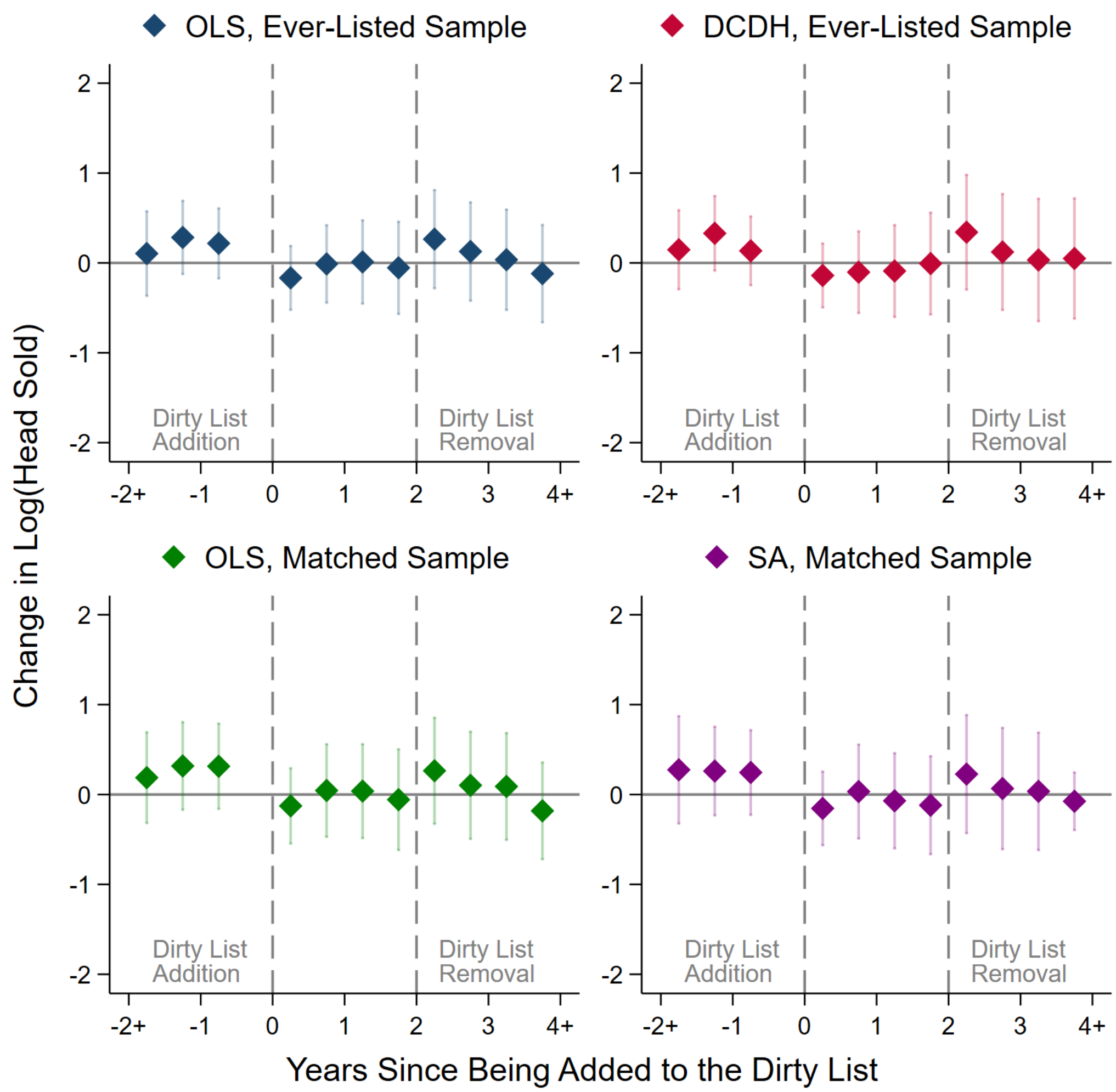

Figure S10: Changes in indirect sales of younger animals (<24 months) over time

Bars represent 95% confidence intervals. Reference period is one semester prior to Dirty List addition. Results presented from testing changes in sales to other ranchers of younger, non-slaughter-age animals over time using four different specifications: OLS with rancher and period fixed effects on the sample of ever-Dirty-Listed ranchers (robustness specification); the de Chaisemartin-D'Haultfœuille (DCDH) estimator on the sample of ever-Dirty-Listed ranchers (preferred specification); OLS with rancher and period fixed effects on the "matched sample" consisting of a subset of ever-listed ranchers plus matched never-listed ranchers (robustness specification); and the Sun-Abraham (SA) estimator on the matched sample (robustness specification).

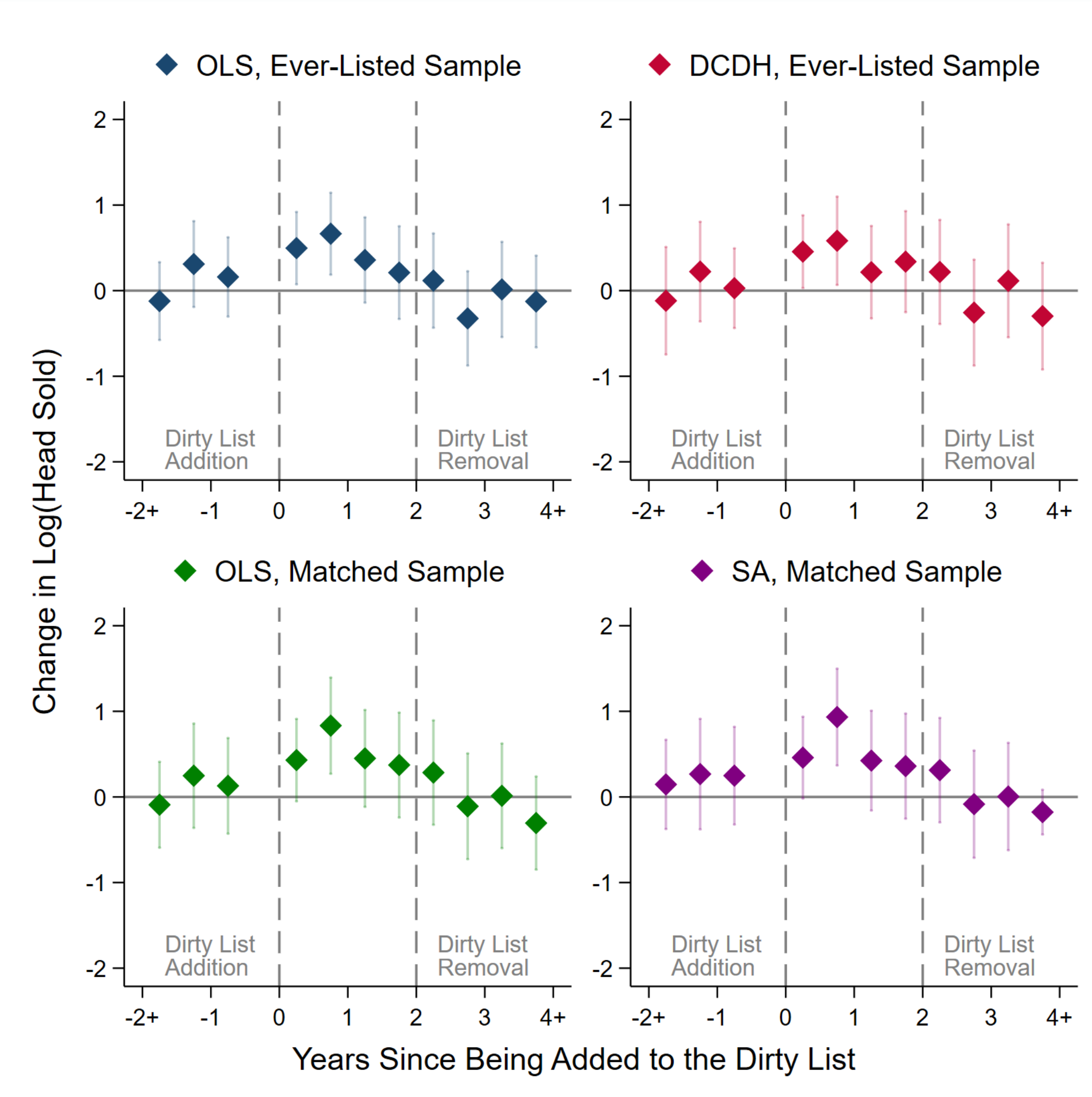

Figure S11: Changes in indirect sales of slaughter-age animals (>24 months) over time

Bars represent 95% confidence intervals. Reference period is one semester prior to Dirty List addition. Results presented from testing changes in sales to other ranchers of mature, slaughter-age animals over time using four different specifications: OLS with rancher and period fixed effects on the sample of ever-Dirty-Listed ranchers (robustness specification); the de Chaisemartin-D'Haultfœuille (DCDH) estimator on the sample of ever-Dirty-Listed ranchers (preferred specification); OLS with rancher and period fixed effects on the "matched sample" consisting of a subset of ever-listed ranchers plus matched never-listed ranchers (robustness specification); and the Sun-Abraham (SA) estimator on the matched sample (robustness specification).

Supplemental Tables

Table S1: Ranchers in Samples, by State

	Main Sample	Matched Sample		
State	DL Total	Not DL	DL	Total
MT	95	61	54	115
PA	163	124	154	278
RO	25	58	20	78
TO	49	30	47	77
Total	332	273	275	548

Note: "DL" refers to ranchers who ever appeared on the Dirty List and "not DL" refers to ranchers who never appeared on the Dirty List. Our primary specifications rely on a sample of 332 ranchers who ever appeared on the Dirty List. For robustness, we also ran our primary specifications with a matched sample consisting of a subset of ranchers who appeared on the Dirty List at some point and for whom we had sufficient data to match on, as well as matched ranchers who were never listed.

Table S2: Number of GTA Transaction Records in DL (Main) Sample, by State and Year

Year	MT	PA	RO	TO	Total
2009	5,767	0	2,746	0	8,513
	(3.4)	(0.0)	(1.6)	(0.0)	(5.0)
2010	6,256	0	3,027	0	9,283
	(3.7)	(0.0)	(1.8)	(0.0)	(5.5)
2011	5,339	0	2,638	0	7,977
	(3.2)	(0.0)	(1.6)	(0.0)	(4.7)
2012	4,197	0	2,585	0	6,782
	(2.5)	(0.0)	(1.5)	(0.0)	(4.0)
2013	4,737	7,199	2,337	0	14,273
	(2.8)	(4.3)	(1.4)	(0.0)	(8.4)
2014	3,896	7,589	1,281	0	12,766
	(2.3)	(4.5)	(0.8)	(0.0)	(7.6)
2015	4,101	6,385	1,871	0	12,357
	(2.4)	(3.8)	(1.1)	(0.0)	(7.3)
2016	4,062	8,569	1,728	0	14,359
	(2.4)	(5.1)	(1.0)	(0.0)	(8.5)
2017	5,552	8,591	1,415	4,692	20,250
	(3.3)	(5.1)	(0.8)	(2.8)	(12.0)
2018	5,519	8,026	0	4,662	18,207
	(3.3)	(4.7)	(0.0)	(2.8)	(10.8)
2019	0	6,015	0	2,948	8,963
	(0.0)	(3.6)	(0.0)	(1.7)	(5.3)
2020	0	6,554	0	3,736	10,290
	(0.0)	(3.9)	(0.0)	(2.2)	(6.1)
2021	0	5,506	0	3,838	9,344
	(0.0)	(3.3)	(0.0)	(2.3)	(5.5)
2022	0	5,142	0	3,557	8,699
	(0.0)	(3.0)	(0.0)	(2.1)	(5.1)
2023	0	2,880	0	4,041	6,921
	(0.0)	(1.7)	(0.0)	(2.4)	(4.1)
Total	49,426	72,456	19,628	27,474	168,984
	(29.2)	(42.9)	(11.6)	(16.3)	(100.0)

Note: Percent of total transaction records in parentheses. GTA data availability varies by state and year

Table S3: Number of GTA Transaction Records in Matched (Robustness) Sample, by State and Year

Year	MT	PA	RO	TO	Total
2009	5,147	0	3,223	0	8,370
	(2.8)	(0.0)	(1.7)	(0.0)	(4.5)
2010	6,008	0	3,525	0	9,533
	(3.2)	(0.0)	(1.9)	(0.0)	(5.2)
2011	4,823	0	3,196	0	8,019
	(2.6)	(0.0)	(1.7)	(0.0)	(4.3)
2012	4,170	0	3,341	0	7,511
	(2.3)	(0.0)	(1.8)	(0.0)	(4.1)
2013	4,456	7,799	3,108	0	15,363
	(2.4)	(4.2)	(1.7)	(0.0)	(8.3)
2014	4,059	8,390	2,001	0	14,450
	(2.2)	(4.5)	(1.1)	(0.0)	(7.8)
2015	4,387	7,109	2,591	0	14,087
	(2.4)	(3.8)	(1.4)	(0.0)	(7.6)
2016	4,258	9,406	2,481	0	16,145
	(2.3)	(5.1)	(1.3)	(0.0)	(8.7)
2017	5,755	9,458	2,095	4,835	22,143
	(3.1)	(5.1)	(1.1)	(2.6)	(12.0)
2018	5,804	8,970	0	4,859	19,633
	(3.1)	(4.8)	(0.0)	(2.6)	(10.6)
2019	0	6,883	0	3,049	9,932
	(0.0)	(3.7)	(0.0)	(1.6)	(5.4)
2020	0	7,449	0	3,980	11,429
	(0.0)	(4.0)	(0.0)	(2.2)	(6.2)
2021	0	6,491	0	4,074	10,565
	(0.0)	(3.5)	(0.0)	(2.2)	(5.7)
2022	0	5,978	0	3,891	9,869
	(0.0)	(3.2)	(0.0)	(2.1)	(5.3)
2023	0	3,478	0	4,433	7,911
	(0.0)	(1.9)	(0.0)	(2.4)	(4.3)
Total	48,867	81,411	25,561	29,121	184,960
	(26.4)	(44.0)	(13.8)	(15.7)	(100.0)

Note: Percent of total transaction records in parentheses. GTA data availability varies by state and year

Table S4: Mean Sales Behavior by a Ranch (number of head sold in 6 Months), by Sales Destination

	Main Sample	Matched Sample		
	Main-DL	Matched-Non-DL	Matched-DL	Matched-Total
All Sales	943	154	1,053	609
	(2,810)	(515)	(3,041)	(2,238)
Direct Sales Only				
All Slaughterhouses	323	50	367	211
	(1,018)	(218)	(1,101)	(813)
Direct CA - PLC	135	22	154	89
	(623)	(153)	(676)	(497)
Direct CA - TAC	173	27	202	116
	(782)	(176)	(851)	(624)
Direct Non-CA	22	10	25	17
Export-Certified	(104)	(68)	(112)	(93)
Direct Non-CA	66	8	74	41
Not Export-Certified	(362)	(56)	(385)	(279)
Indirect Sales Only				
Indirect Heads	620	104	685	398
	(2,132)	(381)	(2,306)	(1,687)
Indirect, Younger Animals	302	55	341	200
(<2 years)	(1,255)	(235)	(1,361)	(992)
Indirect, Older Animals	275	39	309	175
(>2 years)	(1,072)	(179)	(1,158)	(843)
Indirect CA - PLC	113	14	125	70
	(560)	(85)	(603)	(436)
Indirect CA - TAC	176	19	196	109
	(841)	(114)	(905)	(655)
Observations	6502	5293	5412	10,705

Note: Standard deviations in parentheses. Statistics are averages for the entire study period. Our primary specifications rely on a sample of 332 ranchers who ever appeared on the Dirty List. For robustness, we also ran our primary specifications with a matched sample consisting of 278 ranchers who appeared on the Dirty List during our study period and for whom we had sufficient data to match on, as well as 275 matched ranchers who were never listed.

Table S5: Number of Slaughterhouses Connected to Dirty Listed Ranchers, by Sale Destination

	Sale Destination				
	US	UK	EU	China	Other
**Bought from dirty property either directly or indirectly and**					
Exported to ... at any point in study period	1	11	27	19	55
Exported to ... in same year as dirty purchase	0	3	18	4	51
**Bought from dirty property directly and**					
Exported to ... at any point in study period	1	4	13	10	29
Exported to ... in same year as dirty purchase	0	0	4	2	15
**Bought from dirty property indirectly and**					
Exported to ... at any point in study period	1	11	27	19	55
Exported to ... in same year as dirty purchase	0	3	18	3	51

Note: "EU" does not include UK. All counts are likely higher, since export data only cover 2014, 2018, and 2019, while the study period spanned 2009-2024.

Table S6: Robustness Results: Matched Sample Estimation of Changes in Head Sold to Different Destinations

Outcome	Rancher on DL	Rancher Formerly on DL	p-val: On DL = Formerly on DL	Obs	R^2	Mean Sales (Head per Semester)
All Sales	-0.49** (0.24)	-1.01*** (0.26)	0.01**	10705	0.618	609
Direct Sales Only						
All Direct Sales	-1.51*** (0.23)	-1.46*** (0.22)	0.82	10705	0.643	211
Direct CA - PLC	-1.06*** (0.18)	-0.48*** (0.18)	0.00***	10705	0.565	89
Direct CA - TAC	-0.67*** (0.18)	0.03 (0.22)	0.00***	10705	0.558	116
Direct Non-CA - SIF	-0.62*** (0.21)	-0.92*** (0.23)	0.05*	10705	0.419	17
Direct Non-CA - Non-SIF	-0.52*** (0.14)	-0.66*** (0.13)	0.14	10705	0.446	41
Indirect Sales Only						
All Indirect Sales	0.20 (0.23)	-0.53** (0.25)	0.00***	10705	0.529	398
Indirect - Younger (0-24 mo)	-0.18 (0.23)	-0.21 (0.26)	0.85	10705	0.486	200
Indirect - Older (24+ mo)	0.54** (0.22)	-0.08 (0.23)	0.00***	10705	0.438	175
Indirect CA - PLC	0.60*** (0.20)	0.30* (0.16)	0.04**	10705	0.396	70
Indirect CA - TAC	0.38* (0.21)	0.09 (0.20)	0.08*	10705	0.411	109

Note: SEs in parentheses. This table displays robustness results from estimating Model 1 on a sample consisting of 278 ranchers who appeared on the Dirty List during our study period and for whom we had sufficient data to match on, as well as 275 matched ranchers who were never listed. This sample differs from that of our primary specification, which includes 332 ever-listed ranchers and no never-listed ranchers. Estimated coefficients approximately represent the percent change in sales to various destinations during the two years a rancher is on the Dirty List and after being removed, compared to the periods before listing. *$p<0.10$, ** $p<0.05$, *** $p<0.01$.

Table S7: Robustness Results: PPML Estimation of Changes in Head Sold to Different Destinations

Outcome	Rancher on DL	Rancher Formerly on DL	p-val: On DL = Formerly on DL	Obs	Mean Sales (Head per Semester)
All Sales	-0.10<br>(0.11)	-0.33**<br>(0.12)	0.08	6502	943
Direct Sales Only					
All Direct Sales	-0.63***<br>(0.10)	-0.58***<br>(0.11)	0.65	5498	323
Direct CA - PLC	-0.84***<br>(0.08)	-0.58**<br>(0.17)	0.08	2668	135
Direct CA - TAC	-0.89***<br>(0.04)	-0.68***<br>(0.12)	0.00***	4018	173
Direct Non-CA - SIF	-0.32<br>(0.24)	-0.60<br>(0.32)	0.39	3441	22
Direct Non-CA - Non-SIF	-0.61***<br>(0.12)	-0.62***<br>(0.13)	0.91	3995	66
Indirect Sales Only					
All Indirect Sales	0.27*<br>(0.17)	-0.14<br>(0.17)	0.02**	6442	620
Indirect - Younger (0-24 mo)	-0.03<br>(0.25)	-0.12<br>(0.30)	0.66	5891	302
Indirect - Older (24+ mo)	0.52**<br>(0.26)	-0.12<br>(0.20)	0.01***	6259	275
Indirect CA - PLC	0.58<br>(0.47)	-0.16<br>(0.32)	0.02**	4702	113
Indirect CA - TAC	0.07<br>(0.20)	-0.40*<br>(0.18)	0.03**	5534	176

Note: SEs in parentheses. This table displays robustness results from estimating Model 1 using the Poisson pseudo maximum likelihood (PPML) estimator with rancher and period fixed effects [36]. To allow for comparability with coefficients from the log-linear model, we exponentiated estimated PPML coefficients and subtracted one (i.e. performed the transformation $e^{\beta} - 1$). Reported coefficients therefore represent the proportion change in sales to various destinations during the two years a rancher is on the Dirty List and after being removed, compared to the periods before listing. *p$<$0.10, ** p$<$0.05, *** p$<$0.01.

Table S8: Robustness Results: Relationship Between Timing of Deforestation and Dirty Listing

	(1)<br>Ha of Deforestation	(2)<br>Proportion of Holding Deforested
Time Since Dirty Listing		
5+ Years Before	-59.33	-0.000374
	(192.6)	(0.000499)
4 Years Before	-227.6	-0.000679
	(159.1)	(0.000889)
3 Years Before	-120.4	-0.00103
	(178.3)	(0.000640)
2 Years Before	-3.575	-0.000587
	(193.3)	(0.000861)
Year of Listing	-199.3	-0.000957
	(154.8)	(0.000729)
1 Years After	-187.3	-0.00152*
	(153.7)	(0.000845)
2 Years After	-181.4	-0.00146*
	(150.2)	(0.000844)
3 Years After	-179.7	-0.00168**
	(156.0)	(0.000792)
4+ Years After	-191.5	-0.00137*
	(168.3)	(0.000784)
Observations	9813	9813
R^2	0.144	0.143

Note: SEs in parentheses. Results from robustness analysis described in Section **??** to test for correlation between timing of deforestation by Dirty Listed ranchers and timing of Dirty List inclusion. Specification was an event study model regressing deforestation (hectares and proportion of rancher's holding) by rancher r in year t on years until/since Dirty Listing with year and rancher fixed effects. Sample is matched sample including all Dirty Listed ranchers for whom property location information is available plus matches. Results suggest that the timing of deforestation by Dirty Listed ranchers is not significantly correlated with the timing of Dirty List inclusion, which rules out deforestation as a potential confounding driver of our results. *p<0.10, ** p<0.05, *** p<0.01

References

[1] ILO. Global Estimates of Modern Slavery: Forced Labour and Forced Marriage. Technical report, 2022.

[2] ILO. What is forced labour, modern slavery and human trafficking (Forced labour, modern slavery and human trafficking), 2021.

[3] Patricía Trindade Maranhão Costa. *Fighting forced labour: the example of Brazil*. Internat. Labour Off, Geneva, 2009.

[4] Natalia Sayuri Suzuki. *Trabalho escravo contemporâneo: institucionalizações e representações no desenvolvimento da política pública de erradicação*. text, Universidade de São Paulo, April 2023.

[5] Rebecca Scott, Leonardo de Andrade Barbosa, and Carlos Haddad. How Does the Law Put a Historical Analogy to Work?: Defining the Imposition of "A Condition Analogous to That of a Slave" in Modern Brazil. *Duke Journal of Constitutional Law and Public Policy*, 13(1), December 2017.

[6] Tim Höflinger. Brazil's Combat Against Modern Slavery Has An Enforcement Problem. *Forbes*, March 2023. Section: Policy.

[7] Saulo Nogueira and Evgenia Ustinova. Livestock and Products Annual, August 2021.

[8] ABIEC. Beef Report. Technical report, 2024.

[9] Concepta McManus, Júlio Otávio Jardim Barcellos, Bruna Krummenauer Formenton, Potira Meirelles Hermuche, Osmar Abílio de Carvalho Jr, RenatoFontes Guimarães, Miguelangelo Gianezini, Eduardo Antunes Dias, Vinícius do Nascimento Lampert, Daniele Zago, and José Braccini Neto. Dynamics of Cattle Production in Brazil. *PLOS ONE*, 11(1):e0147138, January 2016. Publisher: Public Library of Science.

[10] Silvia Marina Pinheiro, Caroline Emberson, and Alexander Trautrims. 'For the English to see' or effective change? How supply chains are shaped by laws and regulations and what that means for the exposure of modern slavery. *Journal of the British Academy*, 7(s1), June 2019. Number: s1 Publisher: British Academy.

[11] Nicola Phillips and Leonardo Sakamoto. Global Production Networks, Chronic Poverty and 'Slave Labour' in Brazil. *Studies in Comparative International Development*, 47(3):287–315, September 2012. Publisher: Springer Nature.

[12] Daniela Issa. Reification and the Human Commodity: Theorizing Modern Slavery in Brazil. *Latin American Perspectives*, 44(6):90–106, 2017. Publisher: Sage Publications, Inc.

[13] André Campos, Piero Locatelli, and Marcel Gomes. Reporter Brasil Monitor #8 Slave Labor in Brazil's Meat Industry. Technical report, 2021.

[14] Juliana Brandão, Lisa Rausch, Jacob Munger, Lisa Naughton-Treves, and Holly K. Gibbs. Behind the Cattle Industry: Modern Slave Labor Used to Produce Brazil's Beef and Leather. *Environmental Development*, page 101000, May 2024.

[15] Holly K. Gibbs, Jacob Munger, Jessica L'Roe, Paulo Barreto, Ritaumaria Pereira, Matthew Christie, Ticiana Amaral, and Nathalie F. Walker. Did Ranchers and Slaughterhouses Respond to Zero-Deforestation Agreements in the Brazilian Amazon? *Conservation Letters*, 9(1):32–42, 2016. _eprint: https://onlinelibrary.wiley.com/doi/pdf/10.1111/conl.12175.

[16] Marin Elisabeth Skidmore, Fanny Moffette, Lisa Rausch, Matthew Christie, Jacob Munger, and Holly K. Gibbs. Cattle ranchers and deforestation in the brazilian amazon: Production, location, and policies. *Global Environmental Change*, 68:102280, 2021.

[17] Manuela Andreoni, Hiroko Tabuchi, Albert Sun, and Victor Moriyama. How Americans' Appetite for Leather in Luxury SUVs Worsens Amazon Deforestation. *The New York Times*, November 2021.

[18] Erasmus K. H. J. zu Ermgassen, Mairon G. Bastos Lima, Helen Bellfield, Adeline Dontenville, Toby Gardner, Javier Godar, Robert Heilmayr, Rosa Indenbaum, Tiago N. P. dos Reis, Vivian Ribeiro, Itohan-osa Abu, Zoltan Szantoi, and Patrick Meyfroidt. Addressing indirect sourcing in zero deforestation commodity supply chains. *Science Advances*, 8(17):eabn3132, April 2022. Publisher: American Association for the Advancement of Science.

[19] Jennifer Alix-Garcia and Holly K. Gibbs. Forest conservation effects of Brazil's zero deforestation cattle agreements undermined by leakage. *Global Environmental Change*, 47:201–217, November 2017.

[20] Thales A. P. West, Lisa Rausch, Jacob Munger, and Holly K. Gibbs. Protected areas still used to produce Brazil's cattle. *Conservation Letters*, 15(6):e12916, 2022. _eprint: https://onlinelibrary.wiley.com/doi/pdf/10.1111/conl.12916.

[21] Raoni Rajão, Britaldo Soares-Filho, Felipe Nunes, Jan Börner, Lilian Machado, Débora Assis, Amanda Oliveira, Luis Pinto, Vivian Ribeiro, Lisa Rausch, Holly Gibbs, and Danilo Figueira. The rotten apples of Brazil's agribusiness. *Science*, 369(6501):246–248, July 2020.

[22] Environmental Justice Foundation. Trabalho escravo no setor pecuarista: o caso de Mato Grosso e Mato Grosso do Sul. Technical report, 2023.

[23] Ryan Abman, Clark Lundberg, and Michele Ruta. The effectiveness of environmental provisions in regional trade agreements. *Journal of the European Economic Association*, 22(6):2507–2548, 03 2024.

[24] Jennifer M. Alix-Garcia, Katharine R. E. Sims, and Patricia Yañez-Pagans. Only one tree from each seed? environmental effectiveness and poverty alleviation in mexico's payments for ecosystem services program. *American Economic Journal: Economic Policy*, 7(4):1–40, November 2015.

[25] Liyang Sun and Sarah Abraham. Estimating dynamic treatment effects in event studies with heterogeneous treatment effects. *Journal of Econometrics*, 225(2):175–199, December 2021.

[26] INDEA Mato Grosso.

[27] IDARON Rondônia.

[28] ADEPARÁ Pará.

[29] ADAPEC Tocantins.

[30] Amintas Brandão Jr., Lisa Rausch, Jacob Munger, and Holly K. Gibbs. Mapping Slaughterhouse Supply Zones in the Brazilian Amazon with Cattle Transit Records. *Land*, 12(9):1782, September 2023.

[31] Rafael Suzuki Queiroz and Rodrigo Tannus de Queiroz. Carta Boi - A evolução do peso de abate de bovinos - Scot Consultoria, May 2021.

[32] Agrolink. Cotações de Bovinos.

[33] Gary S. Becker. Crime and Punishment: An Economic Approach. *Journal of Political Economy*, 76(2):169–217, 1968. Publisher: University of Chicago Press.

[34] Fanny Moffette, Marin Skidmore, and Holly K. Gibbs. Environmental policies that shape productivity: Evidence from cattle ranching in the Amazon. *Journal of Environmental Economics and Management*, 109:102490, September 2021.

[35] Globo. Justiça condena dono de fazenda a pagar R$ 6 milhões por manter dezenas de trabalhadores em situação de escravidão em MT, October 2018.

[36] Sergio Correia, Paulo Guimarães, and Thomas Zylkin. ppmlhdfe: Fast Poisson Estimation with High-Dimensional Fixed Effects. *The Stata Journal: Promoting communications on statistics and Stata*, 20(1):95–115, March 2020. arXiv:1903.01690 [econ].
